# Implication of heart rate variability on cerebral small vessel disease: A potential therapeutic target

**DOI:** 10.1111/cns.14111

**Published:** 2023-02-14

**Authors:** Yu Tian, Dongxiao Yao, Yuesong Pan, Mengxing Wang, Xia Meng, Xingquan Zhao, Liping Liu, Yongjun Wang, Yilong Wang

**Affiliations:** ^1^ Department of Neurology, Beijing Tiantan Hospital Capital Medical University Beijing China; ^2^ China National Clinical Research Center for Neurological Diseases Beijing China; ^3^ Chinese Institute for Brain Research Beijing China; ^4^ Advanced Innovation Center for Human Brain Protection Capital Medical University Beijing China; ^5^ National Center for Neurological Diseases Beijing China

**Keywords:** autonomic nervous system, cerebral small vessel disease, heart rate variability, white matter hyperintensity

## Abstract

**Objective:**

This study aimed to investigate the relationships of heart rate variability (HRV) with the presence, severity, and individual neuroimaging markers of cerebral small vessel disease (CSVD).

**Method:**

A total of 4676 participants from the Third China National Stroke Registry (CNSR‐III) study were included in this cross‐sectional analysis. CSVD and its markers, including white matter hyperintensity (WMH), lacunes, enlarged perivascular spaces (EPVS), cerebral microbleeds (CMBs), and brain atrophy (BA), were evaluated. Two common HRV parameters, including the square root of the mean of the sum of the squares of differences between adjacent N–N intervals (RMSSD) and the standard deviation of all N–N intervals (SDNN), were used to evaluate the function of the autonomic nervous system (ANS). Binary or ordinal logistic regression analyses were performed to investigate the association between HRV and CSVD. In addition, two‐sample mendelian randomization (MR) analyses were performed to investigate the causality of HRV with CSVD.

**Results:**

RMSSD was significantly associated with total burden of CSVD (Wardlaw's scale, common odds ratio [cOR] 0.80, 95% confidence interval [CI] 0.67–0.96, *p* = 0.02; Rothwell's scale, cOR 0.75, 95% CI 0.60–0.93, *p* = 0.008) and the presence of CSVD (Rothwell, OR 0.75, 95% CI 0.60–0.93, *p* = 0.008). However, no significant associations between SDNN and the presence or total burden of CSVD were observed. Moreover, RMSSD was related to WMH burden (OR 0.80, 95% CI 0.66–0.96, *p* = 0.02), modified WMH burden (cOR 0.82, 95% CI 0.69–0.97, *p* = 0.02), and Deep‐WMH (OR 0.75, 95% CI 0.62–0.91, *p* = 0.003), while SDNN was related to Deep‐WMH (OR 0.80, 95% CI 0.66–0.96, *p* = 0.02) and BA (cOR 0.80, 95% CI 0.68–0.95, *p* = 0.009). Furthermore, adding HRV to the conventional model based on vascualr risk factors enhanced the predictive performance for CSVD, as validated by the integrated discrimination index (*p* < 0.05). In addition, no causality between HRV and CSVD was observed in two‐sample MR analyses.

**Conclusion:**

Decreased HRV may be a potential risk factor of CSVD, implying the possible role of the ANS in the pathogenesis of CSVD.

## Graphical abstract

This study found that decreased heart rate variability parameters, including the square root of the mean of the sum of the squares of differences between adjacent N–N intervals (RMSSD) and the standard deviation of all N–N intervals (SDNN), were partly associated with the presence, severity, and imaging markers of cerebral small vessel disease (CSVD). These findings emphasized a possible role of autonomic nervous dysfunction in the pathogenesis of CSVD, and intervention of the autonomic nervous system may be a novel therapeutic target for CSVD in the future.

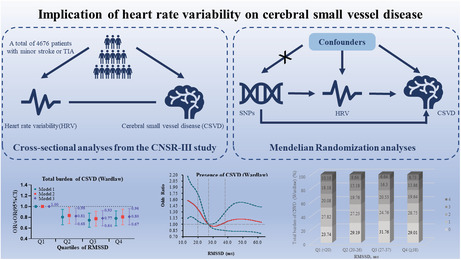

## 1 INTRODUCTION

Cerebral small vessel disease (CSVD) refers to a range of ischemic and micro‐hemorrhagic manifestations involving arterioles, venules, and capillaries.^1^ CSVD is the quarter cause of stroke and the leading cause of vascular cognitive impairment.^2^ Typical neuroimaging features of CSVD on magnetic resonance imaging (MRI) include white matter hyperintensity (WMH), lacunes, enlarged perivascular spaces (EPVS), cerebral microbleeds (CMBs), and brain atrophy (BA).^3^ Previous studies have identified a spectrum of traditional vascular risk factors and blood‐based biomarkers for CSVD.^4^, ^5^, ^6^, ^7^ However, despite abundant literature on this subject, these is insufficient explanation regarding the biological processes of CSVD. Therefore, identifying novel biomarkers for a better understanding of CSVD may provide a strong foundation for advances in knowledge and clinical management.^8^, ^9^

Heart rate variability (HRV), as an index reflecting the balance of the autonomic nervous system (ANS), is related to prognosis of cardiovascular disease.^10^ Moreover, lower HRV is associated with hypertension, diabetes, cognition, emotion, and sleep disorder.^11^, ^12^, ^13^ Despite the growing evidence, there is still a notable gap in literature on the link between HRV and CSVD.^14^ Current observational studies are limited to relatively small samples and incomplete imaging assessments of CSVD to cover the potential role of the ANS in CSVD. To the best of our knowledge, no prior study has investigated associations between HRV and presence and severity of CSVD and its heterogeneous imaging phenotypes. Moreover, observational results are susceptible to confounding and reverse causality bias. Using genetic variants associated with exposures as instruments, mendelian randomization (MR) analysis is more suitable to explore causality between the exposures and CSVD.^15^ A recent advance has provided genetic support for the causal relationship between HRV and WMH^16^; however, it is still insufficient to understand the associations between HRV and several neuroimaging markers of CSVD.

Hence, the present study aims to explore the relationships of HRV with presence, severity, and imaging phenotypes of CSVD, illustrating how the imbalance of the ANS exerts a potential effect on CSVD. To do this, we firstly capitalized on cross‐sectional data from the Third China National Stroke Registry (CNSR‐III) study. Subsequently, we characterized genetic robustness and causalities between HRV and diverse CSVD markers using a two‐sample MR analysis.

## 2 METHODS

### 2.1 Study design and population

**FIGURE 1:**
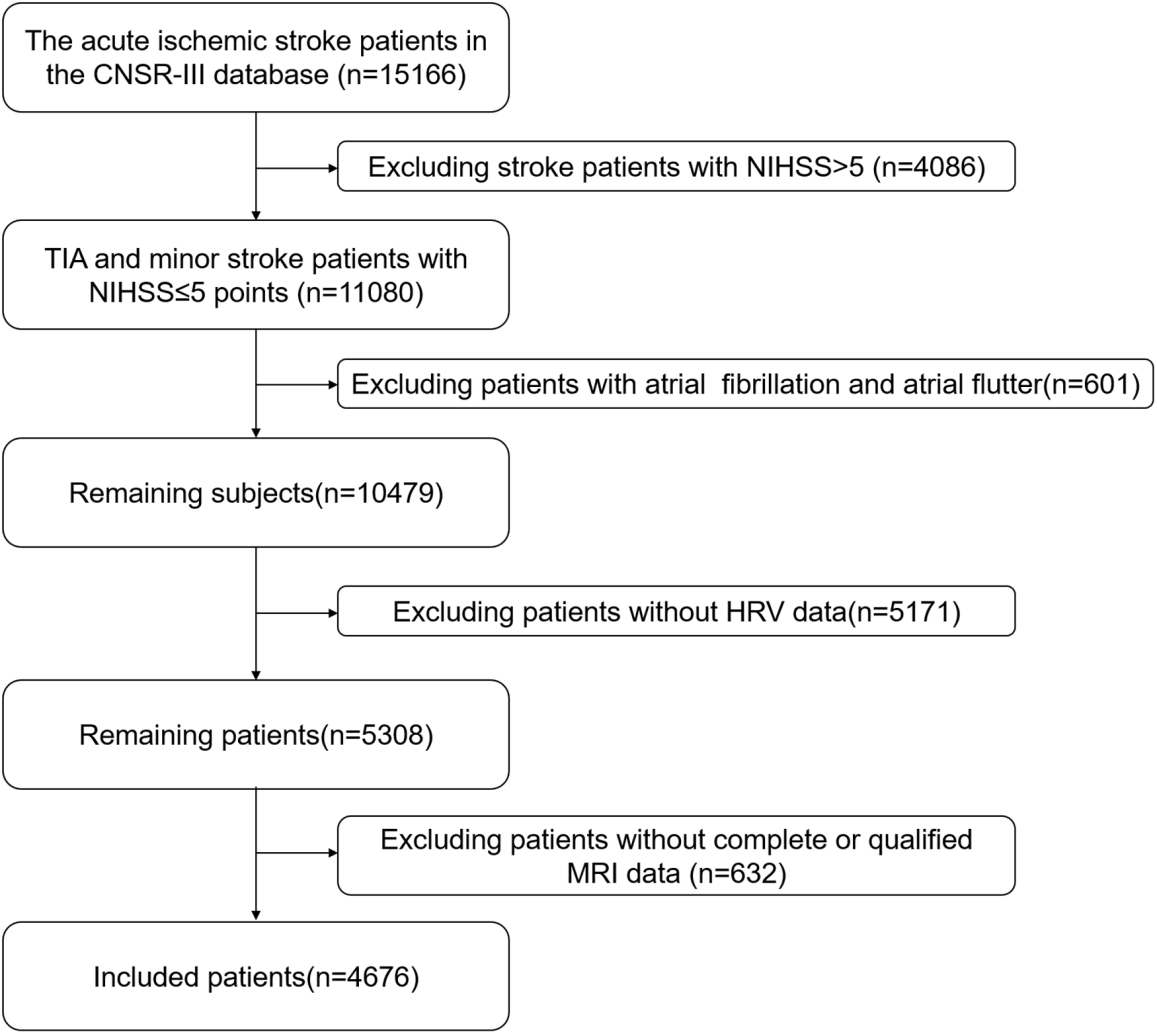
The flowchart of the study. CNSR‐III, the Third China National Stroke Registry; HRV, heart rate variability; TIA, transient ischemic attack.

Data were obtained from the CNSR‐III study. The CNSR‐III study is a multicenter prospective observational clinical cohort in China from August 2015 to March 2018. Details regarding the CNSR‐III study are available in a previous report.^17^ Briefly, 15,166 individuals were enrolled in the CNSR‐III study after they met the following inclusion criteria: (a) age ≥ 18 years, (b) those patients that suffered from an acute ischemic stroke or transient ischemic attack (TIA), and (c) those that were admitted to the hospital within 7 days from the onset of symptoms. We further excluded participants who met the following criteria: (a) 4086 participants with NIHSS scores >5, (b) 601 participants with atrial fibrillation or arrhythmia, (c) 5171 participants without HRV data, and (d) 632 participants without complete or eligible MRI data. Eventually, a total of 4,676 individuals were eligible for the present study (Figure 1).

The CNSR‐III study was conducted in accordance with the guidelines of the Declaration of Helsinki. This study was approved by the Ethics Committee of Beijing Tiantan Hospital (IRB approval number: KY2015‐001‐01) and all the participating centers. All participants or their representatives provided written informed consent prior to enrolment.

### 2.2 Clinical data collection

Trained interviewers prospectively collected clinical data in an electronic data capture system, including demographic characteristics (such as age and sex), medical history (such as stroke/TIA, diabetes mellitus, hypertension, hyperlipidemia, coronary artery disease [CAD]), current smoking and drinking statuses, body mass index (BMI), systolic and diastolic blood pressure (SBP and DPB), heart rate (HR), medications during hospitalization or at discharge (such as antiplatelet treatment, anticoagulation treatment, statin treatment, hypoglycemic treatment, antihypertension treatment, angiotensin‐converting enzyme inhibitor use, angiotensin receptor blocker use, calcium entry blocker use, alpha‐blocker use, and beta‐blocker use), modified Rankin scale (mRS) score, National Institutes of Health Stroke Scale (NIHSS) score, and Trial of Org 10,172 in Acute Stroke Treatment (TOAST) classification.

### 2.3 Measurement of HRV

HRV is a noninvasive method for assessing the balance between the sympathetic (SNS) and parasympathetic nervous system (PNS).^11^ HRVs were generated and analyzed from electrocardiograph (ECG) recordings in participants who received 24‐h Holter monitoring (HS3S3, Xian Landcom Digital Medical Science And Technology Co., Ltd.) within 7 days of hospitalization. All patients were required to participate in regular physical activities during the day and sleep at 10 p.m. at night.

Two most commonly time‐domain HRV indicators were used in this study. The standard deviation of all N–N intervals (SDNN), calculated by the standard deviation of all normal R–R intervals in milliseconds, represented all cyclic components responsible for HRV in all ECG waves.^10^ The SDNN serveed as a comprehensive index to reflect both sympathetic and parasympathetic tones.^13^, ^18^ The square root of the mean of the sum of the squares of differences between adjacent N–N intervals (RMSSD), which mainly reflected the beat‐to‐beat variance in R–R intervals, was solely regulated by the PNS.^13^ Decreased RMSSD was strongly related to impaired vagally mediated changes.^13^, ^18^

### 2.4 Measurement of CSVD

The following structural imaging protocol on a 3.0 Tesla MRI was recommended for all participants: T1‐weighted (T1w), T2‐weighted (T2w), fluid‐attenuated inversion recovery (FLAIR), diffusion‐weighted imaging (DWI) with apparent diffusion coefficient (ADC), and susceptibility‐weighted imaging (SWI) sequences. All MRI data were collected from participating centers in digital format and reviewed by well‐trained readers who were blinded to the clinical data of individuals at Beijing Tiantan Hospital.

All neuroimaging markers of CSVD, including WMH, EPVS, lacunes, CMBs, and BA, were analyzed according to the Standards for Reporting Vascular Changes on Neuroimaging (STRIVE) guidelines.^19^ WMH, as increased signals on FLAIR, was separately assessed in periventricular (PV‐WMH) and deep white matter (D‐WMH) according to the Fazekas scale from 0 to 3.^20^ The WMH burden was defined as PV‐WMH Fazekas 3 or D‐WMH Fazekas 2–3.^4^ Modified WMH burden was defined as Grade 0: total Fazekas score (PV‐WMH + D‐WMH) 0–2, Grade 1: total Fazekas score 3–4, and Grade 2: total Fazekas score 5–6.^21^ PV‐WMH burden or D‐WMH burden was defined as Fazekas score 2–3.^22^ EPVS were observed as small punctate or linear structures of CSF intensity on T1w, T2w, and FLAIR in the basal ganglia (BG‐EPVS) and centrum semiovale (CSO‐EPVS).^23^ The severity of EPVS was counted separately at the level of CSO or BG as none =0, mild = 1–10, moderate = 11–20, frequent = 21–40, and severe = >40 per side on the T2w sequence, and the worse of the two sides was used if there was asymmetry.^24^ We further classified both BG‐EPVS and CSO‐EPVS into three categories: Grade 0: none‐to‐mild, Grade 1: moderate, and Grade 2: frequent‐to‐severe.^25^ Lacunes were defined as ovoid or rounded lesions with a diameter of >3 or < 20 mm and a CSF signal intensity on T1w and T2w, always with a hyperintense rim on FLAIR. CMBs were defined as hypointense round or ovoid lesions within the brain parenchyma with clear margins ranging from 2–15 mm in diameter on SWI/T2*. We described both number and location of CMBs. CMB burden was defined as grade 0: 0, grade 1: 1–4, and grade 2: ≥5 according to the counted number. Strict lobar CMBs were defined as CMBs located only in lobar regions (including frontal, parietal, temporal, and occipital); and non‐strict CMBs were defined as CMBs located in deep (including the basal ganglia and thalamus, corpus callosum) or infratentorial (including the brain stem and cerebellum) regions, with or without lobar CMBs.^26^ BA was estimated using global cortical atrophy (GCA) scale.

The presence and burden of CSVD were evaluated based on a validated CSVD score designed by Wardlaw et al.^4^ and a modified CSVD score described by Rothwell et al.,^21^ respectively. On Wardlaw's scale (from 0 to 4), one point was awarded for each of the following: (a) WMH burden, (b) presence of lacunes, (c) presence of CMBs, and (d) moderate‐to‐severe BG‐EPVS. On Rothwell's scale (from 0 to 6), one point was awarded on each of the following: (a) modified WMH burden Grade 1, (b) presence of lacunes, (c) CMBs burden Grade 1, and (d) frequent‐to‐severe BG‐EPVS; and two points were awarded on each of the following: (e) CMB burden Grade 2, (f) modified WMH burden Grade 2. If total burden was 0 points, CSVD was considered absent; otherwise, it was considered present. Moderate‐to‐severe CSVD was defined as Wardlaw's scale 2 to 4 points and Rothwell's scale 2 to 6 points, respectively.

### 2.5 Statistics analysis

The included participants were divided into four groups according to the quartiles of HRV index according to previous studies.^27^, ^28^, ^29^ Continuous variables were assessed for normal distribution (Kolmogorov–Smirnov test). Continuous variables with normal distribution were described as means with standard deviations (SD), and one‐way ANOVA tests were used to compare the differences across different groups. Non‐normal continuous variables were reported as medians with interquartile ranges (IQR), and Kruskal–Wallis tests were conducted to compare the differences across different groups. Categorical variables were presented as absolute and relative frequencies, and χ^2^ tests were performed to compare the differences across different groups.

The associations of HRV indicators with the presence, total burden, and imaging features of CSVD were investigated using logistic regression analyses. For the total CSVD burden, modified WMH burden, CMB burden, BG‐EPVS, CSO‐EPVS, and BA, ordinal logistic regression analyses were conducted, and common odds ratios (cORs) with their 95% confidence intervals (CIs) were calculated. For the presence of CSVD, WMH burden, PV‐WMH, D‐WMH, lacune, CMBs, strict lobar CMBs, and non‐strict lobar CMBs, binary logistic regression analyses were performed, and ORs with their 95% CIs were calculated. To eliminate underlying confounding bias, three models were constructed: the first model was not adjusted for any covariates; the second model was adjusted for age and sex; and the last model was adjusted for all confounders at *p* < 0.05 in the univariate analyses, including age, sex, HR, SBP, DBP, current smoker, current drinker, hypertension, diabetes, TIA or stroke, CAD, anti‐hypoglycemic treatment, β‐blocker treatment, NIHSS score, mRS score, and qualifying events.

To capture dose–response relationships of HRV with CSVD, binary logistic regression analyses with restricted cubic splines were performed after adjusting potential confounders in model 3. The 50th percentile of HRV was treated as the reference, and five knots for spline were placed at the 5th, 25th, 50th, 75th, and 95th percentiles of HRV.^30^

In addition, we calculated the integrated discrimination improvement (IDI) and net reclassification index (NRI) to evaluate the predictive ability of adding RMSSD and SDNN to the basic model based on traditional vascular risk factors for CSVD.

The significance level was set at *p* < 0.05 (two‐sided). All statistical analyses were performed using the SAS software (version 9.4; SAS Institute, Inc).

### 2.6 Two‐sample MR analysis

MR analysis, analogous to a randomized controlled trail, uses genetic variants (single‐nucleotide polymorphisms, SNPs) associated with the exposure as instrumental variables to infer the risk of diseases and causality between them.^31^ Since Tian et al. have explored the relationships of HRV parameters with WMH volume and small vessel stroke using two‐sample MR analyses,^16^ we further explored the genetic associations between HRV and white matter integrity based on diffusion tensor imaging (DTI) (including fractional anisotropy [FA] and mean diffusivity [MD]), WMH volume, lacunar stroke, and CMBs (including strict lobar CMBs and non‐strict lobar CMBs). Summary‐level data on the associations of SNPs with HRV and CSVD were obtained from previously published genome‐wide association studies (GWAS).^32^, ^33^, ^34^, ^35^ Two‐sample MR analyses were performed to estimate causal effects of HRV on CSVD. The associations between SNP‐HRV and SNP‐outcomes were assessed using inverse variance‐weighted (IVW) method. Sensitivity analyses, including weighted median, simple mode, simple mode, and MR‐Egger tests, were conducted. All MR analyses were performed with R 4.0.3 (R Development Core Team).

## 3 RESULTS

### 3.1 Participant characteristics

A total of 4676 eligible individuals were included in present study, with a mean age of 61.4 ± 10.7 years, and 3242 (69.3%) patients were men. The clinical characteristics of included and excluded patients are presented in Table S1. Significant differences were observed among the participants with respect to age, BMI, current smoking, current drinking, history of TIA/stroke and CAD, anti‐platelet treatment, anticoagulation treatment, antihypertension treatment, NIHSS score, mRS score, qualifying events, and TOAST classification.

The clinical characteristics of the included participants, according to the quartiles of RMSSD and SDNN, are shown in Tables 1 and 2, respectively. The median (IQR) RMSSD was 27(20‐38)^20‐38^ ms, and the median (IQR) SDNN was 105 (88–126) ms. Compared with the Q1 group of HRV, patients in the other three groups were likely to be older, with a higher proportion of men, be current smoker, current drinker, have a history of hypertension, lower HR, SBP, and DBP, a lower prevalence of diabetes, TIA/stroke, hypoglycemic treatment, β‐blocker treatment, antihypertension treatment, and fewer current minor stroke events.

**TABLE 1:** Baseline characteristics of participants according to the quartiles of RMSSD.

Characteristics	Quartile 1 (<20 ms)	Quartile 2 (20–26 ms)	Quartile 3 (27–37 ms)	Quartile 4 (≥38 ms)	*p* value
Age, years, mean ± SD	61.9 ± 10.0	61.2 ± 10.5	61.3 ± 10.7	61.2 ± 11.7	0.30
BMI, kg/m^2^, mean ± SD	24.7 ± 3.2	25.0 ± 3.4	24.9 ± 3.1	24.8 ± 3.4	0.22
Sex, male (%)	347 (30.7)	357 (31.1)	381 (31.4)	349 (29.4)	0.74
Current smoking, *n* (%)	322 (28.5)	393 (34.2)	437 (36.0)	436 (36.8)	<0.001
Current drinking, *n* (%)	153 (13.6)	200 (17.4)	163 (13.4)	181 (15.3)	0.02
SBP, mm Hg, median (IQR)	149.0 (136.0–165.0)	150.0 (136.5–162.5)	147.5 (134.0–163.0)	147.0 (134.0–162.5)	0.08
DBP, mm Hg, median (IQR)	87.5 (80.0–95.0)	87.5 (80.0–95.5)	86.0 (79.0–95.5)	85.0 (78.5–95.0)	0.03
HR, bpm, median (IQR)	76.0 (70.0–82.0)	75.0 (69.0–80.0)	75.0 (68.0–80.0)	72.0 (66.0–80.0)	<0.001
History of disease, *n* (%)					
Diabetes mellitus	312 (27.6)	268 (23.3)	240 (19.8)	230 (19.4)	<0.001
Hypertension	402 (35.6)	415 (36.2)	480 (39.6)	482 (40.6)	0.03
Hyperlipidemia	85 (7.5)	90 (7.8)	105 (8.7)	100 (8.4)	0.74
Stroke/TIA	263 (23.3)	279 (24.3)	266 (21.9)	271 (22.9)	0.59
Coronary artery disease	115 (10.2)	99 (8.6)	132 (10.9)	115 (9.7)	0.31
Medication use during hospitalization or at discharge, *n* (%)					
Anti‐platelet treatment	1115 (98.8)	1139 (99.2)	1204 (99.3)	1179 (99.4)	0.35
Anticoagulation treatment	70 (6.2)	63 (5.5)	72 (5.9)	67 (5.7)	0.89
Stain treatment	1090 (96.6)	1122 (97.7)	1174 (96.8)	1160 (97.8)	0.15
Hypoglycemic treatment	362 (32.1)	313 (27.3)	282 (23.3)	268 (22.6)	<0.001
Antihypertension treatment	668 (59.2)	637 (55.5)	635 (52.4)	637 (53.7)	0.007
CCB	535 (47.4)	510 (44.4)	505 (41.6)	503 (42.4)	0.03
ACEI/ARB	297 (26.3)	259 (22.6)	275 (22.7)	260 (21.9)	0.06
Diuretic	40 (3.5)	38 (3.3)	27 (2.2)	35 (3.0)	0.26
α‐blocker	2 (0.2)	4 (0.4)	2 (0.2)	1 (0.1)	0.52
β‐blocker	57 (5.1)	41 (3.6)	44 (3.6)	49 (4.1)	0.25
NIHSS on admission, median (IQR)	2.0 (1.0–4.0)	2.0 (1.0–4.0)	2.0 (1.0–4.0)	2.0 (1.0–4.0)	0.17
mRS, median (IQR)	0.0 (0.0–0.0)	0.0 (0.0–0.0)	0.0 (0.0–0.0)	0.0 (0.0–1.0)	0.03
Qualifying event, *n* (%)					0.10
MS	1028 (91.1)	1025 (89.3)	1116 (92.0)	1087 (91.7)	
TIA	101 (9.0)	123 (10.7)	97 (8.0)	99 (8.4)	
TOAST, *n*(%)					0.63
Large artery atherosclerosis	268 (23.7)	297 (25.9)	296 (24.4)	322 (27.2)	
Cardioembolism	21 (1.9)	14 (1.2)	20 (1.7)	13 (1.1)	
Small vascular occlusion	288 (25.5)	306 (26.7)	333 (27.5)	298 (25.1)	
Other determination	18 (1.6)	19 (1.7)	15 (1.2)	16 (1.4)	
Undetermined	534 (47.3)	512 (44.6)	549 (45.3)	537 (45.3)	

Abbreviations: ACEI, angiotensin‐converting enzyme inhibitors; ARB, angiotensin receptor blocker; BMI, body mass index; CCB, calcium entry blocker; DBP, diastolic blood pressure; HR, heart rate; mRS, modified Rankin scale; MS, minor stroke; NIHSS, National Institutes of Health Stroke Scale score; RMSSD, the square root of the mean of the sum of the squares of differences between adjacent N–N intervals; SBP, systolic blood pressure; TIA, transient ischaemic attack; TOAST, Trial of Org 10,172 in Acute Stroke Treatment.

**TABLE 2:** Baseline characteristics of participants according to the quartiles of SDNN.

Characteristics	Quartile 1 (<88 ms)	Quartile 2 (88–104 ms)	Quartile 3 (105–125 ms)	Quartile 4 (≥126 ms)	*p* value
Age, years, mean ± SD	63.2 ± 11.0	62.01 ± 10.50	60.5 ± 10.2	59.9 ± 10.9	<0.001
BMI, kg/m^2^, mean ± SD	24.7 ± 3.3	24.9 ± 3.5	25.0 ± 3.2	24.8 ± 3.2	0.14
Sex, male (%)	708 (61.3)	765 (67.5)	864 (71.0)	905 (77.4)	<0.001
Current smoking, *n* (%)	304 (26.3)	378 (33.3)	452 (37.1)	454 (38.8)	<0.001
Current drinking, *n* (%)	131 (11.3)	163 (14.4)	185 (15.2)	218 (18.7)	<0.001
SBP, mm Hg, median (IQR)	150.0 (137.5–163.5)	148.5 (134.5–163.5)	149.0 (135.0–163.0)	146.0 (132.5–162.5)	0.01
DBP, mm Hg, median (IQR)	87.0 (80.0–95.0)	86.5 (79.5–95.0)	87.0 (80.0–96.0)	85.5 (79.0–95.0)	0.28
HR, bpm, median (IQR)	76.0 (70.0–82.0)	76.0 (70.0–80.0)	75.0 (68.0–80.0)	72.0 (65.0–79.0)	<0.001
History of disease, *n* (%)					
Diabetes mellitus	341 (29.5)	244 (21.5)	250 (20.5)	215 (18.4)	<0.001
Hypertension	736 (63.7)	711 (62.7)	744 (61.1)	706 (60.4)	0.35
Hyperlipidemia	83 (7.2)	100 (8.8)	101 (8.3)	96 (8.2)	0.54
Stroke/TIA	299 (25.9)	251 (22.1)	254 (20.9)	275 (23.5)	0.03
Coronary artery disease	136 (11.8)	113 (10.0)	107 (8.8)	105 (9.0)	0.06
Medication use during hospitalization or at discharge, *n* (%)					
Anti‐platelet treatment	1142 (98.8)	1127 (99.4)	1206 (99.1)	1162 (99.4)	0.32
Anticoagulation treatment	77 (6.7)	74 (6.5)	69 (5.7)	52 (4.5)	0.09
Stain treatment	1126 (97.4)	1106 (97.5)	1183 (97.2)	1131 (96.8)	0.68
Hypoglycemic treatment	396 (34.3)	303 (26.7)	290 (23.8)	236 (20.2)	<0.001
Antihypertension treatment	662 (57.3)	629 (55.5)	666 (54.7)	620 (53.0)	0.23
CCB	519 (44.9)	513 (45.2)	539 (44.3)	482 (41.2)	0.19
ACEI/ARB	291 (25.2)	260 (22.9)	268 (22.0)	272 (23.3)	0.34
Diuretic	32 (2.8)	34 (3.0)	34 (2.8)	40 (3.4)	0.78
α‐blocker	3 (0.3)	4 (0.4)	1 (0.1)	1 (0.1)	0.35
β‐blocker	67 (5.8)	44 (3.9)	44 (3.6)	36 (3.1)	0.006
NIHSS on admission, median (IQR)	2.0 (1.0–4.0)	2.0 (1.0–4.0)	2.00 (1.0–4.0)	2.0 (1.0–3.0)	<0.001
mRS, median (IQR)	0.0 (0.0–0.0)	0.0 (0.0–0.0)	0.00 (0.0–0.0)	0.0 (0.0–0.0)	0.64
Qualifying event, n (%)					<0.001
MS	1079 (93.3)	1044 (92.1)	1109 (91.1)	1024 (87.6)	
TIA	77 (6.7)	90 (7.9)	108 (8.9)	145 (12.4)	
TOAST, *n* (%)					0.19
Large artery atherosclerosis	320 (27.7)	280 (24.7)	295 (24.2)	288 (24.6)	
Cardioembolism	17 (1.5)	17 (1.5)	16 (1.3)	18 (1.5)	
Small vascular occlusion	281 (24.3)	299 (26.4)	337 (27.7)	308 (26.4)	
Other determination	26 (2.3)	17 (1.5)	16 (1.3)	9 (0.8)	
Undetermined	512 (44.3)	521 (45.9)	553 (45.4)	546 (46.7)	

Abbreviations: ACEI, angiotensin‐converting enzyme inhibitors; ARB, angiotensin receptor blocker; BMI, body mass index; CCB, calcium entry blocker; DBP, diastolic blood pressure; HR, heart rate; mRS, modified Rankin scale; MS, minor stroke; NIHSS, National Institutes of Health Stroke Scale score; SBP, systolic blood pressure; SDNN, the standard deviation of all N–N intervals; TIA, transient ischaemic attack; TOAST, Trial of Org 10,172 in Acute Stroke Treatment.

### 3.2 Associations of HRV with the presence and severity of CSVD

The proportions of moderate‐to‐severe CSVD (Wardlaw) in the quartiles of RMSSD from 1st to 4th were 48.44%, 43.58%, 43.47%, and 42.23%, respectively, and that of moderate‐to‐severe CSVD (Wardlaw) in the quartiles of SDNN from 1st to 4th were 47.01%, 45.05%, 44.18%, and 41.22%, respectively. Details about the proportions of CSVD markers in the quartiles of RMSSD and SDNN are shown in Figures S1 and S2, respectively.

**FIGURE 2:**
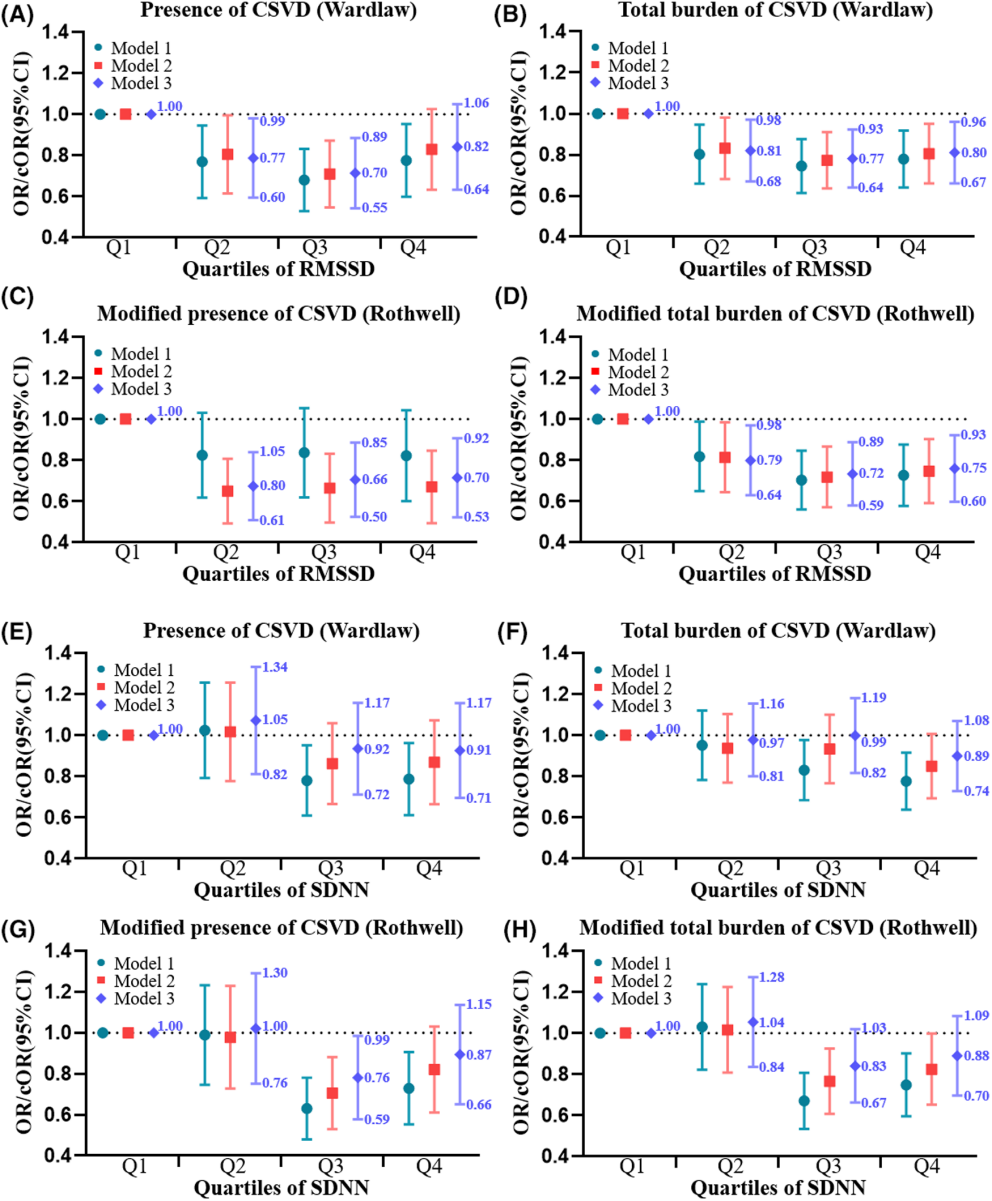
Forest plots for the associations of HRV parameters with CSVD. Forest plots show the ORs/cORs for (A) presence of CSVD (Wardlaw), (B) modified presence of CSVD (Rothwell), (C) total burden of CSVD (Wardlaw), and (D) modified total burden of CSVD (Rothwell) according to the quartiles of RMSSD, and (E) presence of CSVD (Wardlaw), (F) modified presence of CSVD (Rothwell), (G) total burden of CSVD (Wardlaw), and (H) modified total burden of CSVD (Rothwell) according to the quartiles of SDNN, respectively. Association for ordinal categorical outcome of total burden of CSVD was expressed as cOR using ordinal logistic regression, whereas presence of CSVD was expressed as OR using binary logistic regression. The colorful lines represent the 95% confidence intervals of ORs/cORs. Model 1: unadjusted; Model 2: adjusted for age and sex; Model 3: adjusted for adjusted for age, sex, SBP, DBP, HR, current smoker, current drinker, hypertension, diabetes, TIA or stroke, CAD, antihypoglycemic treatment, β‐blocker treatment, NIHSS score, mRS score, and qualifying events. RMSSD, the square root of the mean of the sum of the squares of differences between adjacent N–N intervals; SDNN, the standard deviation of all N–N intervals; CSVD, cerebral small vessel disease; ORs, odds ratios; cORs, common ORs; 95% CI, 95% confidence intervals; SBP, systolic blood pressure; DBP, diastolic blood pressure; HR, heart rate; TIA, transient ischemic attack; CAD, coronary artery disease; NIHSS, National Institutes of Health Stroke Scale score; mRS, modified Rankin scale.

**FIGURE 3:**
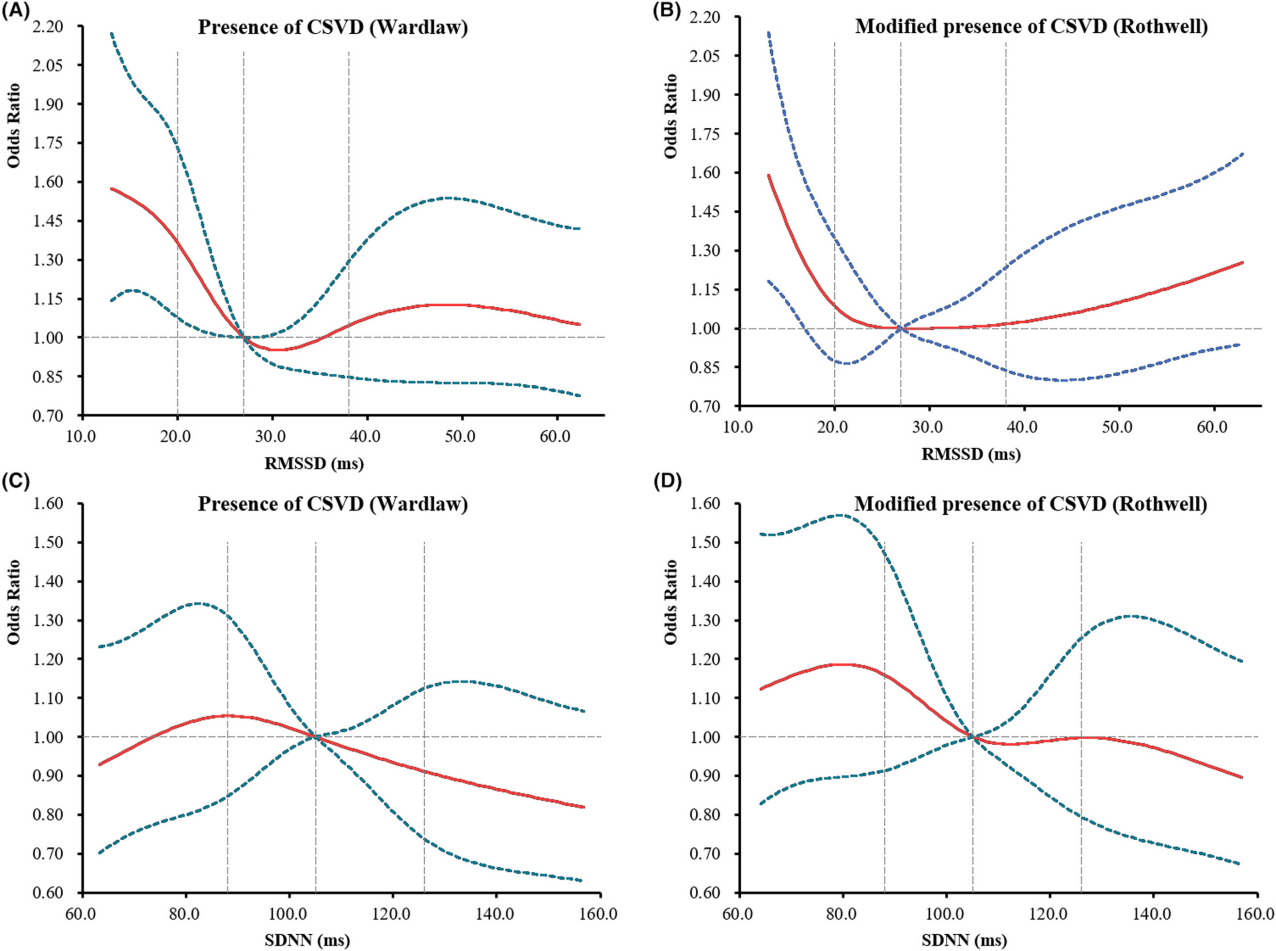
Dose–response associations of HRV parameters with CSVD using restricted cubic splines. Adjusted binary logistic regression models of restricted cubic splines show ORs for (A) presence of CSVD (Wardlaw), (B) modified presence of CSVD (Rothwell) according to RMSSD, and (C) presence of CSVD (Wardlaw), and (D) modified presence of CSVD (Rothwell) according to SDNN, respectively. The solid red line indicates adjusted ORs, and the dashed green lines indicate the 95% CI bands. The reference points are the medians of RMSSD (27 ms) and SDNN(105 ms), respectively. The vertical dashed lines indicate the 25th, 50th, and 75th percentiles of RMSSD and SDNN, respectively. Data were fitted using a logistic regression model of restricted cubic spline with five knots (the 5th, 25th, 50th, 75th, and 95th percentiles) for RMSSD and SDNN after adjusted for age, sex, SBP, DBP, HR, current smoker, current drinker, hypertension, diabetes, TIA or stroke, CAD, antihypoglycemic treatment, β‐blocker treatment, NIHSS score, mRS score, and qualifying events, respectively. The lowest 5% and highest 5% of participants are not shown. RMSSD, the square root of the mean of the sum of the squares of differences between adjacent N–N intervals; SDNN, the standard deviation of all N–N intervals; CSVD, cerebral small vessel disease; ORs, odds ratios; 95% CI, 95% confidence intervals; SBP, systolic blood pressure; DBP, diastolic blood pressure; HR, heart rate; TIA, transient ischemic attack; CAD, coronary artery disease; NIHSS, National Institutes of Health Stroke Scale score; mRS, modified Rankin scale.

After adjusting for all potential confounders, the presence of CSVD (Rothwell) was found to be lower for patients in the Q4 of RMSSD than for those in Q1 (OR 0.75, 95% CI 0.60–0.93, *p* = 0.008; Table S2, Figure 2). Moreover, participants in the Q4 of RMSSD presented a lower total CSVD burden than those in Q1 (Wardlaw: cOR 0.80, 95% CI 0.67–0.96, *p* = 0.02; Rothwell: cOR 0.75, 95% CI 0.60–0.93, *p* = 0.008). Using multivariable binary logistic regression models with restricted cubic splines, L‐shaped associations between RMSSD and the presence and modified presence of CSVD were found (Figure 3).

No statistically significant associations between SDNN and the presence or total burden of CSVD were observed after adjusting for all potential covariates (Table S3, Figures 2 and 3). Moreover, there were non‐linear relationships between SDNN and the presence and modified presence of CSVD (Figure 3).

### 3.3 Associations of HRV with imaging phenotypes of CSVD

**FIGURE 4:**
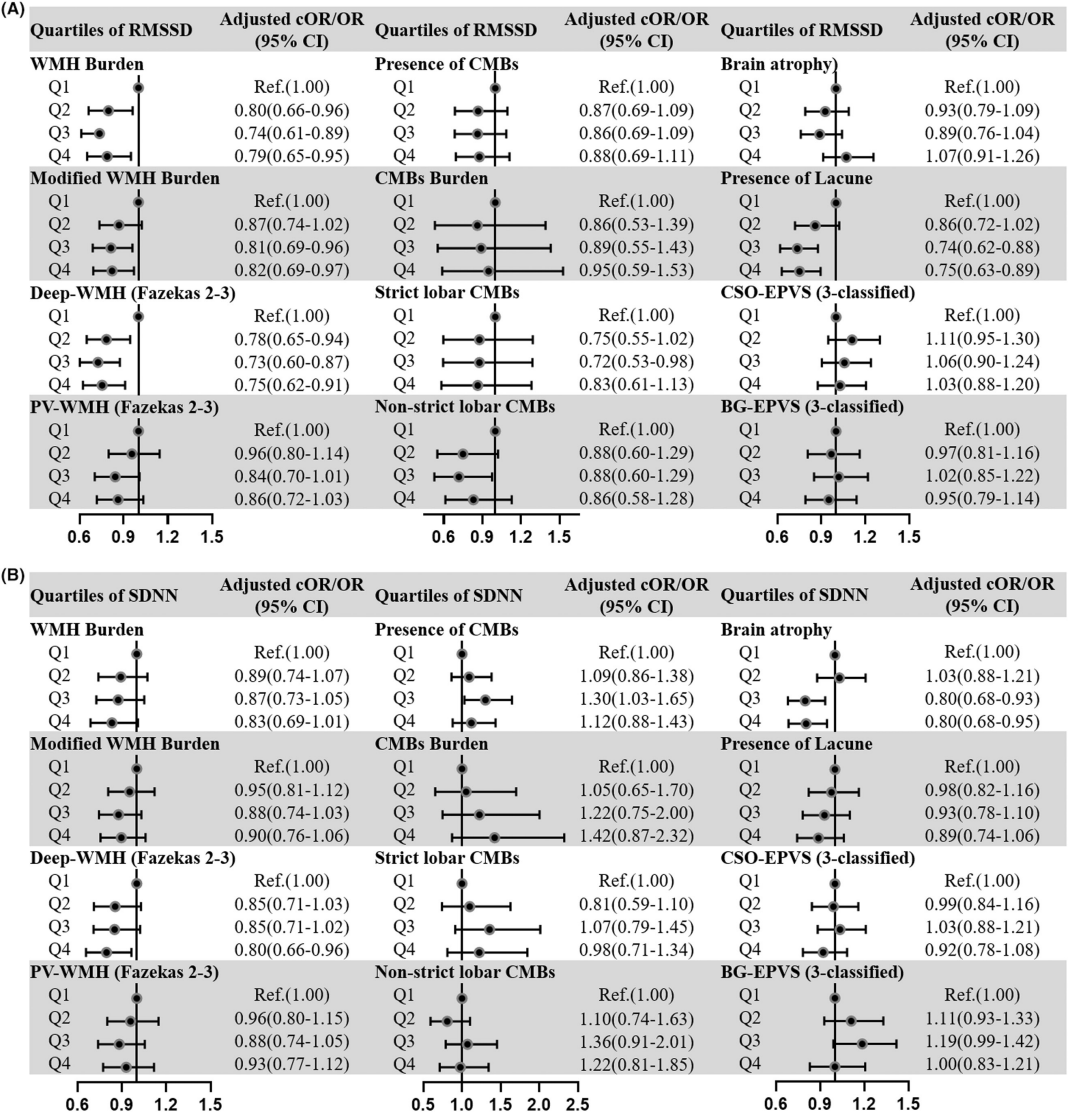
Forest plots for the associations of HRV parameters with CSVD markers. Forest plots show the ORs/cORs for CSVD markers according to the quartiles of (A) RMSSD and (B) SDNN after adjusted for age, sex, SBP, DBP, HR, current smoker, current drinker, hypertension, diabetes, TIA or stroke, CAD, antihypoglycemic treatment, β‐blocker treatment, NIHSS score, mRS score, and qualifying events. The binary logistic regression analysis was conducted to investigate the association between HRV and imaging markers of CSVD, including WMH burden, D‐WMH, PV‐WMH, lacunes, CMBs, strict lobar CMBs, non‐strict lobar CMBs, and brain atrophy. The ORs and 95% CI were calculated. The ordinal logistic regression analysis was conducted to investigate the association between HRV and imaging markers of CSVD, including modified WMH burden, CMBs burden, BG‐EPVS, and CSO‐EPVS. The cORs and 95% CI were calculated. RMSSD, the square root of the mean of the sum of the squares of differences between adjacent N–N intervals; SDNN, the standard deviation of all N–N intervals; CSVD, cerebral small vessel disease; WMH, white matter hyperintensity; PV‐WMH, periventricular WMH; D‐WMH, deep‐WMH; CMBs, cerebral microbleeds; EPVS, enlarged perivascular spaces; BG‐EPVS, basal ganglia EPVS; CSO‐EPVS, centrum semiovale EPVS; GCA, global brain atrophy; cOR, common odd ratio; OR, odd ratio; 95% CI, 95% confidence intervals; SBP, systolic blood pressure; DBP, diastolic blood pressure; HR, heart rate; TIA, transient ischemic attack; CAD, coronary artery disease; NIHSS, National Institutes of Health Stroke Scale score; mRS, modified Rankin scale.

Q4 of RMSSD was related to WMH burden (OR 0.80, 95% CI 0.66–0.96, *p* = 0.02), modified WMH burden (cOR 0.82, 95% CI 0.69–0.97, *p* = 0.02), and D‐WMH burden (OR 0.75, 95% CI 0.62–0.91, *p* = 0.003), but not to PV‐WMH burden (Figure 4; Table S4). A lower risk of lacunes was observed in Q4 of RMSSD than in Q1 (OR 0.75, 95% CI 0.63–0.89, *p* = 0.001). There were no significant associations between RMSSD and CMBs, BG‐ and CSO‐EPVS, and BA persisted.

Compared to Q1, Q4 of SDNN was associated with D‐WMH (OR 0.80, 95% CI 0.66–0.96, *p* = 0.02) and BA (cOR 0.80, 95% CI 0.68–0.95, *p* = 0.009; Figure 4; Table S5). There were no significant differences between SDNN and the other CSVD imaging markers, including WMH burden, modified WMH burden, PV‐WMH, CMBs, BG‐ and CSO‐EPVS.

### 3.4 Incremental predictive models of HRV for CSVD

We estimated whether the addition of HRV parameters would further increase the predictive values of the traditional model based on several vascular risk factors on CSVD, as validated by IDIs and NRIs (Table S6). The IDIs of individual SDNN and individual RMSSD, as well as combined SDNN and RMSSD for the presence of CSVD (Rothwell) were 0.16% (0.01%, 0.30%), 0.18% (0.01%, 0.35%), and 0.24% (0.05%, 0.44%), respectively.

### 3.5 Genetically predicted association between HRV and CSVD

The characteristics of the GWASs used in this study are shown in Table S7, and the characteristics of selected SNPs of HRV are shown in Table S8. No genetic associations of HRV with WMH volume, FA, MD, lacunar stroke, and CMBs were described in two‐sample MR analyses (Table S9).

## 4 DISCUSSION

In this study, we found that HRV was significantly associated with the risk of CSVD, especially total burden, WMH, lacunes, and BA, in patients with minor stroke/TIA. Notably, RMSSD showed more substantial relationships with the presence, severity, and heterogeneous imaging features of CSVD than SDNN, except for cortical atrophy. These results implied that dysfunction of the ANS as denoted by low HRV parameters may be a nonconventional risk factor for CSVD.

Because of diverse HRV parameters and heterogeneous imaging characteristics of CSVD, the link between HRV and CSVD was investigated incomprehensively, and previous results were difficult to compare. Two common HRV parameters, that could fully reflect the function of the ANS, were selected for this study. Our results indicated that a lower RMSSD, a parasympathetic‐mediated HRV indicator, was associated with severity of WMH, especially D‐WMH. This was in line with previous reports that decreased RMSSD was associated with white matter lesions.^36^, ^37^ A lower RMSSD also presented a relationship with lacunes in our current analysis. Interestingly, abnormal blood pressure response to the Valsalva test was related to neurological progression after acute lacunar infarction, implying the potential role of ANS function in predicting neurological progression of lacunar stroke.^38^ To the best of our knowledge, our study is the first to find that the presence and total burden of CSVD were significantly related to decreased RMSSD, highlighting the role of parasympathetic overactivity in CSVD. Inconsistent to our results, a previous report showed that higher nighttime RMSSD as estimated using ambulatory blood pressure monitoring (ABPM) was a predictor of the progression of CSVD and WMH.^39^ Several points should be taken into account in this contradiction. Firstly, the clinical appliance of HRV in ABPM was less clear than with 24‐h Holter utilized in our study. The 24‐h Holter had an established widespread clinical utility, such as quantifying autonomic function via power spectral analysis of HRV, and was deemed as the gold standard method of HR assessment.^40^ Secondly, it remained unclear that whether HR measured every other hour accompanied by intermittent cuff inflation could reflect true individual HRV level.^41^, ^42^ Hence, to some extent, we should interpret this positive relationship between RMSSD and CSVD with cautions.

However, our data differed somewhat from previous studies for the controversial association of SDNN—an overall HRV index reflecting joint sympathetic and parasympathetic balance—with CSVD. Only D‐WMH and BA were associated with lower SDNN in the present study. No significant differences in SDNN were found in older patients classified by D‐WMH and PV‐WMH, respectively.^43^ Moreover, no significant relationship between SDNN and WMH was observed in patients with cognitive dysfunction.^36^ However, recent advances showed that only nighttime SDNN, but not daytime HRV, was adversely associated with WMH and CSVD.^37^, ^44^, ^45^, ^46^ One possibility was that different designs of the studies resulted in these inconsistencies. HRV during the awake period was usually found to be unstable because participants were supposed to perform physical activities and experience emotional triggers that may affect balance of the ANS. Furthermore, the association between SDNN and BA was also supported by a recent report in which SDNN was positively associated with gray matter volume in the right inferior frontal gyrus of patients with CSVD.^44^

To further illustrate the potential effect of HRV on CSVD, two‐sample MR analysis was performed to investigate the causality. Our data differed somewhat from a previous report for the causal relationship between RMSSD and WMH.^13^ In fact, DTI‐derived metrics, including decreased FA and increased MD, were more sensitive to microstructural alterations of white matter than WMH volume, which only appeared under pathological conditions.^47^ The differences in stages of damage to white matter integrity may be attributed to these paradoxical results. However, genetic variants associated with these traits explained only a small fraction of variation in risk factors. Insufficient statistical power may result in a lack of significant relationships of HRV with FA and MD.

As there was no previous evidence on the associations between HRV and CMBs and EPVS, as far as we concerned, our results did not find any significant associations in either our observational study or MR analysis.

Although genetically predicted causality between HRV and CSVD was still considered controversial, we, at the very least, provided observational evidence that declined HRV was a potential risk factor of CSVD. More importantly, the addition of HRV parameters to the basic risk model showed an incremental effect on the predictive ability for incident CSVD, implying the need for increased exploitation of HRV in risk stratification and medical strategy of CSVD. Using HRV, especially the RMSSD‐related parameters that represent the activity of the PNS, could help neurologists to identify accurately individuals with high risk of CSVD that deserved more attention and required additional vigilance. Declined HRV, as a potential untraditional risk factor, may provide a clue that was different from the information reflected by neuroimaging features or/and other traditional vascular risk factors. More researches are needed into the research field of using other HRV values to predict the incidence and severity of CSVD, and, therefore, the future consequences of CSVD, such as cognitive decline. If our present findings were verified in different CSVD‐related population, they may provide relevant information for the clinical care and risk stratification of patients with CSVD. Additionally, we speculated that specific interventions targeted toward the dysfunction of the ANS as reflected by decreased HRV, such as vagus nerve stimulation, may reduce the risk of occurrence of CSVD and delay the development of CSVD. A recent rodent study showed nicotinamide riboside (NR) administration alleviated angiotensin II (Ang‐II)–induced CSVD via protecting blood–brain barrier integrity, vascular remodeling, and neuroinflammation.^48^ Given the links between Ang‐II and decreased HRV, enhanced activity of the ANS may mediate the treatment effect of NR on CSVD.^49^ We considered the hypothesis that ANS dysfunction could be a potential therapeutic target of CSVD. Future longitudinal studies are required to clarify this theory in the elderly population or in patients with CSVD.

The underlying mechanisms of the association between CSVD and ANS were not well‐elucidated; however, several possibilities were put forth as follows. Firstly, increased mechanical stress on the endothelium and subsequent endothelial injury induced by the imbalance between sympathetic and parasympathetic tones were presumed to contribute to the failure of dynamic cerebral autoregulation and blood–brain barrier disruption.^50^, ^51^, ^52^ Secondly, the cerebrovascular architecture in the deep brain parenchyma lacked anastomoses; as a result, these regions were more susceptible to vasodilatory disturbances in rigid vessels caused by abnormal autonomic function.^53^ Thirdly, many risk factors and mechanisms which were reported to be associated with HRV, such as diabetes and inflammation, were deemed to exert interactive effects that enhance the link between HRV and CSVD.^4^, ^6^, ^12^, ^54^ Meanwhile, symptoms including postural hypotension, cognitive impairment, and sleep disorders were related to both HRV and CSVD.^11^, ^55^, ^56^, ^57^ Furthermore, heart rate control may also be a possible reason.^12^ Taken together, the complex series of cascading responses in the regulation of the ANS during CSVD requires further investigation.

This study had several limitations. Firstly, the clinical design of the CNSR‐III cohort included only patients with acute ischemic stroke or TIA. Although we excluded patients with NIHSS scores >5, who were more likely to have large artery atherosclerosis rather than small vessel disease, the aforementioned criteria prevented generalizability of our findings. Therefore, we suggested that this study should be replicated in a large population sample of elderly patients and patients with CSVD. Secondly, our present findings were based on cross‐sectional investigation, and it did not allow for causal inferences between HRV and the progression of CSVD and imaging markers. Although MR analyses were performed to explore the causal effects of HRV and CSVD, no significant associations were found in the present study. Thirdly, our study included only Chinese population (enrolled in the CNSR‐III study), which limited generalization to other ethnic groups. However, we adopted GWAS data in two‐sample MR analyses, which were derived from participants of European ancestry. Differences in ethnicity may result in inconsistent results. Fourthly, only two 24‐hour time‐domain HRV parameters (RMSSD and SDNN) were investigated. The role of other HRV parameters, such as the frequency domain and non‐linear parameters, should be extensively explored in CSVD. Moreover, the 24‐hour HRV failed to detect differences between daytime and nighttime, although HRV measured by Holter was accepted as the international standard. Additionally, despite our efforts to adjust for possible covariates in three models, the inherent shortcoming of observational research was that unmeasured confounding factors of HRV and CSVD were inevitable. Lastly, prediction models were only detected in the discovery sample, and more external validation studies are necessary to estimate the predictive power.

## 5 CONCLUSION

We found that decreased HRV parameters, including RMSSD and SDNN, were partly associated with the presence, severity, and imaging markers of CSVD, particularly WMH, lacunes, and BA. These findings emphasized a possible role of the activity of the ANS in the pathogenesis of CSVD, and ANS intervention may be a novel therapeutic target for CSVD in the future.

## AUTHOR CONTRIBUTIONS

YLW and YJW designed the study. MXW and YSP analyzed the data and contributed to reviewing the statistical problems. DXY performed two‐sample MR analyses. YT interpreted the data and drafted the manuscript. YLW reviewed the manuscript. All authors approved the final version of the manuscript.

## FUNDING INFORMATION

This work was supported by grants from National Natural Science Foundation of China (No. 81825007), Beijing Outstanding Young Scientist Program (No. BJJWZYJH01201910025030), Youth Beijing Scholar Program (No. 010), Beijing Talent Project ‐ Class A: Innovation and Development (No. 2018A12), “National Ten‐Thousand Talent Plan”‐ Leadership of Scientific and Technological Innovation, Chinese Academy of Medical Sciences Innovation Fund for Medical Sciences (No. 2019‐I2M‐5‐029), and Capital's Funds for Health Improvement and Research (No. 2020‐1‐2041).

## CONFLICT OF INTEREST STATEMENT

The author(s) declared no potential conflicts of interest with respect to the research, authorship, and/or publication of this article.

## Supporting information


**Data S1:** Supporting information

## Data Availability

All data generated or analyzed during this study are included in this published article and available upon reasonable requests.
