# Supplementary material for: Implication of heart rate variability on cerebral small vessel disease: A potential therapeutic target
Source: CNS Neurosci Ther. 2023 Feb 14;29(5):1379–91. doi: 10.1111/cns.14111 (PMC10068455; doi:10.1111/cns.14111)

**Supplementary Table 1. Baseline Characteristics of included and excluded participants**

| **Characteristics** | **All(n=15166)** | **Included(n=4676)** | **Excluded(n=10490)** | **P value** |
| --- | --- | --- | --- | --- |
| Age, years, mean±SD | 62.3±11.3 | 62.6±11.5 | 61.4±10.7 | <0.001 |
| BMI, kg/m^2^, mean±SD | 24.7±3.3 | 24.6±3.4 | 24.9±3.3 | <0.001 |
| Sex, male(%) | 10364(68.3) | 7122(67.9) | 3242(69.3) | 0.08 |
| Current smoking, n (%) | 4752(31.3) | 3164(30.2) | 1588(34.0) | <0.001 |
| Current drinking, n (%) | 2126(14.0) | 1429(13.6) | 697(14.9) | 0.04 |
| SBP, mm Hg, median (IQR) | 148.0(135.0-136.5) | 148.0(135.0-163.5) | 148.0(135.0-163.3) | 0.56 |
| DBP, mm Hg, median (IQR) | 86.0(79.0-95.0) | 86.0(79.0-95.0) | 86.5(79.5-95.0) | 0.11 |
| HR, bpm, median (IQR) | 75.0(68.0-80.0) | 75.0(68.0-80.0) | 75.0(68.0-80.0) | 0.08 |
| History of disease, n (%) |  |  |  |  |
| Diabetes mellitus | 3510(23.1) | 2460(23.5) | 1050(22.5) | 0.18 |
| Hypertension | 9494(62.6) | 6597(62.9) | 2897(62.0) | 0.27 |
| Hyperlipidemia | 1191(7.9) | 811(7.7) | 380(8.1) | 0.40 |
| Stroke/TIA | 3675(24.2) | 2596(24.8) | 1079(23.1) | 0.03 |
| Coronary artery disease | 1608(10.6) | 1147(10.9) | 461(9.9) | 0.047 |
| Medication use during hospitalization or at discharge, n(%) |  |  |  |  |
| Anti-platelet treatment | 14822(97.7) | 10185(97.1) | 4637(99.2) | <0.001 |
| Anticoagulation treatment | 1621(10.7) | 1349(12.9) | 272(5.8) | <0.001 |
| Stains treatment | 14731(97.1) | 10185(97.1) | 4546(97.2) | 0.66 |
| Hypoglycemic treatment | 4046(26.7) | 2821(26.9) | 1225(26.2) | 0.37 |
| Antihypertension treatment | 8136(53.7) | 5559(53.0) | 2577(55.1) | 0.02 |
| CCB | 6228(41.1) | 4175(39.8) | 2053(43.9) | <0.001 |
| ACEI/ARB | 3401(22.4) | 2310(22.0) | 1091(23.3) | 0.07 |
| Diuretic | 510(3.4) | 370(3.5) | 140(3.0) | 0.09 |
| α-blocker | 44(0.3) | 35(0.3) | 9(0.2) | <0.001 |
| β-blocker |  | 614(5.9) | 191(4.1) | <0.001 |
| NIHSS on admission, median(IQR) | 3.0(1.0-6.0) | 4.0(2.0-7.0) | 2.0(1.0-4.0) | <0.001 |
| mRS, median(IQR) | 0.0(0.0-1.0) | 0.0(0.0-1.0) | 0.0(0.0-0.0) | <0.001 |
| Qualifying event, n(%) |  |  |  | <0.001 |
| MS | 14146(93.3) | 9890(94.3) | 4256(91.0) |  |
| TIA | 1020(6.7) | 600(5.7) | 420(9.0) |  |
| TOAST, n(%) |  |  |  | <0.001 |
| Large artery atherosclerosis | 3856(25.4) | 2673(25.5) | 1183(25.3) |  |
| Cardioembolism | 917(6.1) | 849(8.1) | 68(1.5) |  |
| Small vascular occlusion | 3165(20.9) | 1940(18.5) | 1225(26.2) |  |
| Other determination | 182(1.2) | 114(1.1) | 68(1.5) |  |
| Undetermined | 7046(46.5) | 4914(46.8) | 2132(45.6) |  |

BMI, body mass index; SBP, systolic blood pressure; DBP, diastolic blood pressure; HR, heart rate; CCB, calcium entry blocker; ACEI, angiotensin-converting enzyme inhibitors; ARB, angiotensin receptor blocker; MS, minor stroke; TIA, transient ischaemic attack; NIHSS, National Institutes of Health Stroke Scale score; mRS, modified Rankin scale; TOAST, Trial of Org 10,172 in Acute Stroke Treatment.

**Supplementary Table 2. Associations of RMSSD with presence and total burden of CSVD**

| **Variables** | **Model 1** | | **Model 2** | | **Model 3** |  |
| --- | --- | --- | --- | --- | --- | --- |
|  | **cOR/OR (95% CI)** | **P value** | **cOR/OR (95% CI)** | **P value** | **cOR/OR (95% CI)** | **P value** |
| **Presence of CSVD (Wardlaw)** | | | | | | |
| Q1 (<20) | Ref.(1.00) |  | Ref.(1.00) |  | Ref.(1.00) |  |
| Q2 (20-26) | 0.76(0.60-0.95) | 0.02 | 0.79(0.62-1.00) | 0.051 | 0.77(0.60-0.99) | 0.04 |
| Q3 (27-37) | 0.67(0.54-0.84) | <0.001 | 0.70(0.55-0.88) | 0.002 | 0.70(0.55-0.89) | 0.004 |
| Q4 (≥38) | 0.76(0.61-0.96) | 0.02 | 0.81(0.64-1.03) | 0.09 | 0.82(0.64-1.06) | 0.13 |
| **Total CSVD burden (Wardlaw)** | | | | | | |
| Q1 (<20) | Ref.(1.00) |  | Ref.(1.00) |  | Ref.(1.00) |  |
| Q2 (20-26) | 0.79(0.66-0.95) | 0.01 | 0.82(0.69-0.99) | 0.03 | 0.81(0.68-0.98) | 0.03 |
| Q3 (27-37) | 0.74(0.62-0.88) | <0.001 | 0.76(0.64-0.91) | 0.003 | 0.77(0.64-0.93) | 0.005 |
| Q4 (≥38) | 0.77(0.64-0.92) | 0.004 | 0.80(0.67-0.96) | 0.01 | 0.80(0.67-0.96) | 0.02 |
| **Modified presence of CSVD (Rothwell)** | | | | | | |
| Q1 (<20) | Ref.(1.00) |  | Ref.(1.00) |  | Ref.(1.00) |  |
| Q2 (20-26) | 0.81(0.63-1.04) | 0.10 | 0.82(0.63-1.06) | 0.13 | 0.80(0.61-1.05) | 0.11 |
| Q3 (27-37) | 0.64(0.50-0.81) | <0.001 | 0.65(0.51-0.84) | <0.001 | 0.66(0.50-0.85) | 0.002 |
| Q4 (≥38) | 0.66(0.52-0.85) | 0.001 | 0.69(0.53-0.89) | 0.005 | 0.70(0.53-0.92) | 0.009 |
| **Modified total CSVD burden (Rothwell)** | | | | | | |
| Q1 (<20) | Ref.(1.00) |  | Ref.(1.00) |  | Ref.(1.00) |  |
| Q2 (20-26) | 0.81(0.66-0.99) | 0.04 | 0.80(0.65-0.99) | 0.04 | 0.79(0.64-0.98) | 0.03 |
| Q3 (27-37) | 0.69(0.57-0.85) | <0.001 | 0.71(0.58-0.87) | 0.001 | 0.72(0.59-0.89) | 0.003 |
| Q4 (≥38) | 0.72(0.58-0.88) | 0.002 | 0.74(0.60-0.91) | 0.004 | 0.75(0.60-0.93) | 0.008 |

RMSSD, the square root of the mean of the sum of the squares of differences between adjacent N-N intervals; CSVD, cerebral small vessel disease; cOR, common odd ratio; OR, odd ratio; 95% CI, 95% confidence intervals; SBP, systolic blood pressure; DBP, diastolic blood pressure; HR, heart rate; TIA, transient ischaemic attack; CAD, coronary artery disease; NIHSS, National Institutes of Health Stroke Scale score; mRS, modified Rankin scale.

The binary logistic regression analysis was conducted to investigate the association between RMSSD and presence of CSVD. The ordinal logistic regression analysis was conducted to investigate the association between RMSSD and total burden of CSVD.

Model 1: unadjusted; Model 2: adjusted for age and sex; Model 3: adjusted for age, sex, SBP, DBP, HR, current smoker, current drinker, hypertension, diabetes, TIA or stroke, CAD, antihypoglycemic treatment, β-blocker treatment, NIHSS score, mRS score, and qualifying events.

**Supplementary Table 3. Associations of SDNN with presence and total burden of CSVD**

| **Variables** | **Model 1** | | **Model 2** | | **Model 3** |  |
| --- | --- | --- | --- | --- | --- | --- |
|  | **cOR/OR (95% CI)** | **P value** | **cOR/OR (95% CI)** | **P value** | **cOR/OR (95% CI)** | **P value** |
| **Presence of CSVD (Wardlaw)** | | | | | | |
| Q1 (<80) | Ref.(1.00) |  | Ref.(1.00) |  |  |  |
| Q2 (88-104) | 1.01(0.80-1.27) | 0.95 | 1.00(0.79-1.27) | 0.99 | 1.05(0.82-1.34) | 0.69 |
| Q3 (105-125) | 0.77(0.62-0.96) | 0.02 | 0.85(0.67-1.07) | 0.16 | 0.92(0.72-1.17) | 0.48 |
| Q4 (≥126) | 0.77(0.62-0.97) | 0.03 | 0.85(0.67-1.08) | 0.19 | 0.91(0.71-1.17) | 0.45 |
| **Total CSVD burden (Wardlaw)** | | | | | | |
| Q1 (<80) | Ref.(1.00) |  | Ref.(1.00) |  | Ref.(1.00) |  |
| Q2 (88-104) | 0.94(0.79-1.13) | 0.51 | 0.93(0.78-1.11) | 0.41 | 0.97(0.81-1.16) | 0.72 |
| Q3 (105-125) | 0.82(0.69-0.98) | 0.03 | 0.92(0.77-1.11) | 0.39 | 0.99(0.82-1.19) | 0.90 |
| Q4 (≥126) | 0.77(0.64-0.92) | 0.004 | 0.84(0.70-1.01) | 0.06 | 0.89(0.74-1.08) | 0.22 |
| **Modified presence of CSVD (Rothwell)** | | | | | | |
| Q1 (<80) | Ref.(1.00) |  | Ref.(1.00) |  | Ref.(1.00) |  |
| Q2 (88-104) | 0.97(0.76-1.24) | 0.80 | 0.96(0.74-1.24) | 0.74 | 1.00(0.76-1.30) | 0.99 |
| Q3 (105-125) | 0.62(0.49-0.79) | <0.001 | 0.69(0.54-0.89) | 0.004 | 0.76(0.59-0.99) | 0.04 |
| Q4 (≥126) | 0.72(0.56-0.91) | 0.007 | 0.80(0.62-1.04) | 0.09 | 0.87(0.66-1.15) | 0.33 |
| **Modified total CSVD burden (Rothwell)** | | | | | | |
| Q1 (<80) | Ref.(1.00) |  | Ref.(1.00) |  | Ref.(1.00) |  |
| Q2 (88-104) | 1.02(0.83-1.25) | 0.88 | 1.00(0.82-1.23) | 0.98 | 1.04(0.84-1.28) | 0.72 |
| Q3 (105-125) | 0.66(0.54-0.81) | <0.001 | 0.76(0.61-0.93) | 0.008 | 0.83(0.67-1.03) | 0.08 |
| Q4 (≥126) | 0.74(0.60-0.91) | 0.004 | 0.81(0.66-1.00) | 0.054 | 0.88(0.70-1.09) | 0.23 |

SDNN, the standard deviation of all N-N intervals; CSVD, cerebral small vessel disease; cOR, common odd ratio; OR, odd ratio; 95% CI, 95% confidence intervals; SBP, systolic blood pressure; DBP, diastolic blood pressure; HR, heart rate; TIA, transient ischaemic attack; CAD, coronary artery disease; NIHSS, National Institutes of Health Stroke Scale score; mRS, modified Rankin scale.

The binary logistic regression analysis was conducted to investigate the association between RMSSD and presence of CSVD. The ordinal logistic regression analysis was conducted to investigate the association between RMSSD and total burden of CSVD.

Model 1：unadjusted; Model 2：adjusted for age and sex; Model 3：for adjusted for age, sex, SBP, DBP, HR, current smoker, current drinker, hypertension, diabetes, TIA or stroke, CAD, antihypoglycemic treatment, β-blocker treatment, NIHSS score, mRS score, and qualifying events.

**Supplementary Table 4. Associations of RMSSD with different imaging markers of CSVD**

| **Variables** | **Model 1** | | **Model 2** | | **Model 3** | |
| --- | --- | --- | --- | --- | --- | --- |
|  | **cOR/OR (95% CI)** | **P value** | **cOR/OR (95% CI)** | **P value** | **cOR/OR (95% CI)** | **P value** |
| **WMH Burden** | | | | | | |
| Q1 (<20) | Ref.(1.00) |  | Ref.(1.00) |  | Ref.(1.00) |  |
| Q2 (20-26) | 0.79(0.66-0.93) | 0.006 | 0.80(0.67-0.95) | 0.01 | 0.80(0.66-0.96) | 0.02 |
| Q3 (27-37) | 0.74(0.62-0.88) | <0.001 | 0.74(0.62-0.88) | <0.001 | 0.74(0.61-0.89) | 0.001 |
| Q4 (≥38) | 0.78(0.66-0.93) | 0.004 | 0.78(0.65-0.93) | 0.005 | 0.79(0.65-0.95) | 0.01 |
| **Modified WMH Burden** | | | | | | |
| Q1 (<20) | Ref.(1.00) |  | Ref.(1.00) |  | Ref.(1.00) |  |
| Q2 (20-26) | 0.86(0.74-1.01) | 0.06 | 0.88(0.75-1.03) | 0.1075 | 0.87(0.74-1.02) | 0.091 |
| Q3 (27-37) | 0.82(0.71-0.96) | 0.01 | 0.83(0.70-0.97) | 0.02 | 0.81(0.69-0.96) | 0.01 |
| Q4 (≥38) | 0.84(0.72-0.98) | 0.03 | 0.83(0.71-0.97) | 0.02 | 0.82(0.69-0.97) | 0.02 |
| **PV-WMH (Fazekas 2-3)** | | | | | | |
| Q1 (<20) | Ref.(1.00) |  | Ref.(1.00) |  | Ref.(1.00) |  |
| Q2 (20-26) | 0.92(0.78-1.09) | 0.32 | 0.95(0.80-1.14) | 0.59 | 0.96(0.80-1.14) | 0.62 |
| Q3 (27-37) | 0.83(0.71-0.98) | 0.03 | 0.85(0.71-1.01) | 0.06 | 0.84(0.70-1.01) | 0.06 |
| Q4 (≥38) | 0.85(0.72-1.01) | 0.06 | 0.86(0.73-1.03) | 0.10 | 0.86(0.72-1.03) | 0.11 |
| **Deep-WMH (Fazekas 2-3)** | | | | | | |
| Q1 (<20) | Ref.(1.00) |  | Ref.(1.00) |  | Ref.(1.00) |  |
| Q2 (20-26) | 0.77(0.65-0.92) | 0.003 | 0.78(0.65-0.94) | 0.007 | 0.78(0.65-0.94) | 0.01 |
| Q3 (27-37) | 0.73(0.62-0.87) | <0.001 | 0.73(0.61-0.87) | <0.001 | 0.73(0.60-0.87) | <0.001 |
| Q4 (≥38) | 0.76(0.64-0.90) | 0.001 | 0.75(0.62-0.89) | 0.001 | 0.75(0.62-0.91) | 0.003 |
| **Presence of Lacune** | | | | | | |
| Q1 (<20) | Ref.(1.00) |  | Ref.(1.00) |  | Ref.(1.00) |  |
| Q2 (20-26) | 0.87(0.74-1.03) | 0.11 | 0.89(0.75-1.05) | 0.16 | 0.86(0.72-1.02) | 0.08 |
| Q3 (27-37) | 0.75(0.63-0.88) | <0.001 | 0.76(0.64-0.89) | <0.001 | 0.74(0.62-0.88) | <0.001 |
| Q4 (≥38) | 0.77(0.66-0.91) | 0.002 | 0.78(0.66-0.92) | 0.003 | 0.75(0.63-0.89) | 0.001 |
| **Presence of CMBs** | | | | | | |
| Q1 (<20) | Ref.(1.00) |  | Ref.(1.00) |  | Ref.(1.00) |  |
| Q2 (20-26) | 0.87(0.69-1.09) | 0.21 | 0.88(0.70-1.11) | 0.28 | 0.87(0.69-1.09) | 0.23 |
| Q3 (27-37) | 0.84(0.67-1.05) | 0.13 | 0.86(0.69-1.08) | 0.19 | 0.86(0.69-1.09) | 0.21 |
| Q4 (≥38) | 0.86(0.69-1.08) | 0.20 | 0.88(0.70-1.11) | 0.28 | 0.88(0.69-1.11) | 0.28 |
| **CMBs Burden** | | | | | | |
| Q1 (<20) | Ref.(1.00) |  | Ref.(1.00) |  | Ref.(1.00) |  |
| Q2 (20-26) | 0.84(0.53-1.33) | 0.45 | 0.85(0.53-1.35) | 0.49 | 0.86(0.53-1.39) | 0.54 |
| Q3 (27-37) | 0.80(0.51-1.27) | 0.35 | 0.83(0.52-1.32) | 0.43 | 0.89(0.55-1.43) | 0.63 |
| Q4 (≥38) | 0.91(0.58-1.43) | 0.68 | 0.93(0.59-1.47) | 0.76 | 0.95(0.59-1.53) | 0.82 |
| **Strict lobar CMBs** | | | | | | |
| Q1 (<20) | Ref.(1.00) |  | Ref.(1.00) |  | Ref.(1.00) |  |
| Q2 (20-26) | 0.76(0.57-1.03) | 0.08 | 0.78(0.58-1.06) | 0.11 | 0.75(0.55-1.02) | 0.07 |
| Q3 (27-37) | 0.69(0.51-0.93) | 0.01 | 0.72(0.53-0.97) | 0.03 | 0.72(0.53-0.98) | 0.03 |
| Q4 (≥38) | 0.84(0.63-1.12) | 0.22 | 0.86(0.64-1.15) | 0.31 | 0.83(0.61-1.13) | 0.23 |
| **Non-strict lobar CMBs** | | | | | | |
| Q1 (<20) | Ref.(1.00) |  | Ref.(1.00) |  | Ref.(1.00) |  |
| Q2 (20-26) | 0.85(0.59-1.24) | 0.41 | 0.86(0.59-1.25) | 0.43 | 0.88(0.60-1.29) | 0.51 |
| Q3 (27-37) | 0.79(0.55-1.15) | 0.22 | 0.82(0.56-1.18) | 0.28 | 0.88(0.60-1.29) | 0.50 |
| Q4 (≥38) | 0.81(0.55-1.18) | 0.27 | 0.82(0.57-1.20) | 0.32 | 0.86(0.58-1.28) | 0.47 |
| **BG-EPVS (3-classified)** | | | | | | |
| Q1 (<20) | Ref.(1.00) |  | Ref.(1.00) |  | Ref.(1.00) |  |
| Q2 (20-26) | 0.94(0.79-1.12) | 0.47 | 0.98(0.82-1.17) | 0.79 | 0.97(0.81-1.16) | 0.73 |
| Q3 (27-37) | 0.99(0.83-1.17) | 0.88 | 1.02(0.86-1.22) | 0.81 | 1.02(0.85-1.22) | 0.89 |
| Q4 (≥38) | 0.93(0.79-1.11) | 0.43 | 0.95(0.80-1.14) | 0.60 | 0.95(0.79-1.14) | 0.58 |
| **CSO-EPVS (3-classified)** | | | | | | |
| Q1 (<20) | Ref.(1.00) |  | Ref.(1.00) |  | Ref.(1.00) |  |
| Q2 (20-26) | 1.11(0.95-1.30) | 0.18 | 1.12(0.96-1.31) | 0.16 | 1.11(0.95-1.30) | 0.20 |
| Q3 (27-37) | 1.04(0.89-1.21) | 0.66 | 1.04(0.89-1.22) | 0.60 | 1.06(0.90-1.24) | 0.49 |
| Q4 (≥38) | 1.02(0.87-1.19) | 0.86 | 1.02(0.87-1.19) | 0.82 | 1.03(0.88-1.20) | 0.75 |
| **Brain atrophy (GCA scale)** | | | | | | |
| Q1 (<20) | Ref.(1.00) |  | Ref.(1.00) |  | Ref.(1.00) |  |
| Q2 (20-26) | 0.87(0.75-1.02) | 0.08 | 0.91(0.77-1.06) | 0.22 | 0.93(0.79-1.09) | 0.34 |
| Q3 (27-37) | 0.83(0.71-0.97) | 0.02 | 0.86(0.73-1.00) | 0.049 | 0.89(0.76-1.04) | 0.14 |
| Q4 (≥38) | 0.95(0.82-1.11) | 0.51 | 1.01(0.86-1.18) | 0.90 | 1.07(0.91-1.26) | 0.40 |

RMSSD, the square root of the mean of the sum of the squares of differences between adjacent N-N intervals; CSVD, cerebral small vessel disease; WMH, white matter hyperintensity; PV-WMH, periventricular WMH; D-WMH, deep-WMH; CMBs, cerebral microbleeds; EPVS, enlarged perivascular spaces; BG-EPVS, basal ganglia EPVS; CSO-EPVS, centrum semiovale EPVS; GCA, global brain atrophy; cOR, common odd ratio; OR, odd ratio; 95% CI, 95% confidence intervals; SBP, systolic blood pressure; DBP, diastolic blood pressure; HR, heart rate; TIA, transient ischaemic attack; CAD, coronary artery disease; NIHSS, National Institutes of Health Stroke Scale score; mRS, modified Rankin scale.

The binary logistic regression analysis was conducted to investigate the association between RMSSD and imaging markers of CSVD, including WMH burden, D-WMH, PV-WMH, lacunes, CMBs, strict lobar CMBs, non-strict lobar CMBs, and brain atrophy. The ordinal logistic regression analysis was conducted to investigate the association between RMSSD and imaging markers of CSVD, including modified WMH burden, CMBs burden, BG-EPVS, and CSO-EPVS.

Model 1：unadjusted; Model 2：adjusted for age and sex; Model 3：adjusted for age, sex, SBP, DBP, HR, current smoker, current drinker, hypertension, diabetes, TIA or stroke, CAD, antihypoglycemic treatment, β-blocker treatment, NIHSS score, mRS score, and qualifying events.

**Supplementary Table 5. Associations of SDNN with different imaging markers of CSVD**

| **Variables** | **Model 1** | | **Model 2** | | **Model 3** | |
| --- | --- | --- | --- | --- | --- | --- |
|  | **cOR/OR (95% CI)** | **P value** | **cOR/OR (95% CI)** | **P value** | **cOR/OR (95% CI)** | **P value** |
| **WMH Burden** | | | | | | |
| Q1 (<80) | Ref.(1.00) |  | Ref.(1.00) |  | Ref.(1.00) |  |
| Q2 (88-104) | 0.80(0.68-0.95) | 0.01 | 0.85(0.71-1.01) | 0.07 | 0.89(0.74-1.07) | 0.22 |
| Q3 (105-125) | 0.71(0.60-0.84) | <0.001 | 0.82(0.69-0.98) | 0.03 | 0.87(0.73-1.05) | 0.15 |
| Q4 (≥126) | 0.64(0.54-0.76) | <0.001 | 0.75(0.63-0.90) | 0.002 | 0.83(0.69-1.01) | 0.06 |
| **Modified WMH Burden** | | | | | | |
| Q1 (<80) | Ref.(1.00) |  | Ref.(1.00) |  | Ref.(1.00) |  |
| Q2 (88-104) | 0.87(0.75-1.02) | 0.09 | 0.91(0.78-1.07) | 0.26 | 0.95(0.81-1.12) | 0.55 |
| Q3 (105-125) | 0.74(0.63-0.86) | <0.001 | 0.84(0.72-0.99) | 0.04 | 0.88(0.74-1.03) | 0.11 |
| Q4 (≥126) | 0.72(0.61-0.84) | <0.001 | 0.83(0.71-0.98) | 0.03 | 0.90(0.76-1.06) | 0.20 |
| **Modified WMH Burden** | | | | | | |
| Q1 (<80) | Ref.(1.00) |  | Ref.(1.00) |  | Ref.(1.00) |  |
| Q2 (88-104) | 0.87(0.73-1.02) | 0.09 | 0.91(0.77-1.09) | 0.30 | 0.96(0.80-1.15) | 0.64 |
| Q3 (105-125) | 0.74(0.63-0.87) | <0.001 | 0.84(0.71-1.00) | 0.048 | 0.88(0.74-1.05) | 0.17 |
| Q4 (≥126) | 0.74(0.63-0.88) | <0.001 | 0.87(0.73-1.04) | 0.12 | 0.93(0.77-1.12) | 0.43 |
| **Deep-WMH (Fazekas 2-3)** | | | | | | |
| Q1 (<80) | Ref.(1.00) |  | Ref.(1.00) |  | Ref.(1.00) |  |
| Q2 (88-104) | 0.77(0.65-0.92) | 0.003 | 0.81(0.68-0.97) | 0.02 | 0.85(0.71-1.03) | 0.10 |
| Q3 (105-125) | 0.69(0.59-0.82) | <0.001 | 0.80(0.67-0.96) | 0.01 | 0.85(0.71-1.02) | 0.09 |
| Q4 (≥126) | 0.62(0.52-0.73) | <0.001 | 0.72(0.60-0.87) | <0.001 | 0.80(0.66-0.96) | 0.02 |
| **Presence of Lacune** | | | | | | |
| Q1 (<80) | Ref.(1.00) |  | Ref.(1.00) |  | Ref.(1.00) |  |
| Q2 (88-104) | 0.94(0.79-1.10) | 0.43 | 0.94(0.79-1.11) | 0.44 | 0.98(0.82-1.16) | 0.80 |
| Q3 (105-125) | 0.86(0.74-1.02) | 0.08 | 0.88(0.75-1.04) | 0.14 | 0.93(0.78-1.10) | 0.39 |
| Q4 (≥126) | 0.84(0.71-0.99) | 0.03 | 0.85(0.72-1.00) | 0.05 | 0.89(0.74-1.06) | 0.19 |
| **Presence of CMBs** | | | | | | |
| Q1 (<80) | Ref.(1.00) |  | Ref.(1.00) |  | Ref.(1.00) |  |
| Q2 (88-104) | 1.07(0.85-1.34) | 0.59 | 1.07(0.85-1.35) | 0.56 | 1.09(0.86-1.38) | 0.48 |
| Q3 (105-125) | 1.17(0.94-1.47) | 0.17 | 1.24(0.99-1.56) | 0.06 | 1.30(1.03-1.65) | 0.03 |
| Q4 (≥126) | 1.02(0.81-1.28) | 0.90 | 1.07(0.85-1.36) | 0.56 | 1.12(0.88-1.43) | 0.36 |
| **CMB Burden** | | | | | | |
| Q1 (<80) | Ref.(1.00) |  | Ref.(1.00) |  | Ref.(1.00) |  |
| Q2 (88-104) | 1.04(0.66-1.65) | 0.86 | 1.05(0.66-1.68) | 0.83 | 1.05(0.65-1.70) | 0.83 |
| Q3 (105-125) | 0.95(0.60-1.53) | 0.84 | 1.12(0.70-1.81) | 0.64 | 1.22(0.75-2.00) | 0.42 |
| Q4 (≥126) | 1.08(0.68-1.72) | 0.74 | 1.24(0.78-1.99) | 0.37 | 1.42(0.87-2.32) | 0.16 |
| **Strict lobar CMBs** | | | | | | |
| Q1 (<80) | Ref.(1.00) |  | Ref.(1.00) |  | Ref.(1.00) |  |
| Q2 (88-104) | 0.81(0.60-1.08) | 0.15 | 0.81(0.60-1.09) | 0.16 | 0.81(0.59-1.10) | 0.18 |
| Q3 (105-125) | 0.91(0.68-1.22) | 0.54 | 1.02(0.76-1.38) | 0.87 | 1.07(0.79-1.45) | 0.66 |
| Q4 (≥126) | 0.85(0.63-1.14) | 0.28 | 0.94(0.69-1.27) | 0.67 | 0.98(0.71-1.34) | 0.89 |
| **Non-strict lobar CMBs** | | | | | | |
| Q1 (<80) | Ref.(1.00) |  | Ref.(1.00) |  | Ref.(1.00) |  |
| Q2 (88-104) | 1.09(0.75-1.59) | 0.66 | 1.09(0.74-1.59) | 0.66 | 1.10(0.74-1.63) | 0.64 |
| Q3 (105-125) | 1.10(0.76-1.61) | 0.61 | 1.21(0.83-1.77) | 0.33 | 1.36(0.91-2.01) | 0.13 |
| Q4 (≥126) | 1.02(0.69-1.50) | 0.93 | 1.09(0.74-1.62) | 0.67 | 1.22(0.81-1.85) | 0.34 |
| **BG-EPVS (3-classified)** | | | | | | |
| Q1 (<80) | Ref.(1.00) |  | Ref.(1.00) |  | Ref.(1.00) |  |
| Q2 (88-104) | 1.01(0.85-1.19) | 0.95 | 1.08(0.90-1.29) | 0.40 | 1.11(0.93-1.33) | 0.26 |
| Q3 (105-125) | 0.97(0.82-1.15) | 0.72 | 1.14(0.96-1.36) | 0.15 | 1.19(0.99-1.42) | 0.06 |
| Q4 (≥126) | 0.83(0.70-0.99) | 0.04 | 0.96(0.80-1.15) | 0.63 | 1.00(0.83-1.21) | 0.99 |
| **CSO-EPVS (3-classified)** | | | | | | |
| Q1 (<80) | Ref.(1.00) |  | Ref.(1.00) |  | Ref.(1.00) |  |
| Q2 (88-104) | 0.98(0.84-1.15) | 0.82 | 0.99(0.84-1.15) | 0.8592 | 0.99(0.84-1.16) | 0.89 |
| Q3 (105-125) | 1.01(0.87-1.18) | 0.89 | 1.03(0.88-1.20) | 0.7564 | 1.03(0.88-1.21) | 0.68 |
| Q4 (≥126) | 0.91(0.78-1.06) | 0.23 | 0.92(0.78-1.08) | 0.2919 | 0.92(0.78-1.08) | 0.31 |
| **Brain atrophy (GCA scale)** | | | | | | |
| Q1 (<80) | Ref.(1.00) |  | Ref.(1.00) |  | Ref.(1.00) |  |
| Q2 (88-104) | 0.93(0.80-1.09) | 0.38 | 0.99(0.84-1.16) | 0.87 | 1.03(0.88-1.21) | 0.71 |
| Q3 (105-125) | 0.69(0.59-0.80) | <0.001 | 0.76(0.65-0.89) | <0.001 | 0.80(0.68-0.93) | 0.005 |
| Q4 (≥126) | 0.66(0.57-0.77) | <0.001 | 0.76(0.65-0.89) | <0.001 | 0.80(0.68-0.95) | 0.009 |

SDNN, the standard deviation of all N-N intervals; CSVD, cerebral small vessel disease; WMH, white matter hyperintensity; PV-WMH, periventricular WMH; D-WMH, deep-WMH; CMBs, cerebral microbleeds; EPVS, enlarged perivascular spaces; BG-EPVS, basal ganglia EPVS; CSO-EPVS, centrum semiovale EPVS; GCA, global brain atrophy; cOR, common odd ratio; OR, odd ratio; 95% CI, 95% confidence intervals; SBP, systolic blood pressure; DBP, diastolic blood pressure; HR, heart rate; TIA, transient ischaemic attack; CAD, coronary artery disease; NIHSS, National Institutes of Health Stroke Scale score; mRS, modified Rankin scale.

The binary logistic regression analysis was conducted to investigate the association between RMSSD and imaging markers of CSVD, including WMH burden, D-WMH, PV-WMH, lacunes, CMBs, strict lobar CMBs, non-strict lobar CMBs, and brain atrophy. The ordinal logistic regression analysis was conducted to investigate the association between RMSSD and imaging markers of CSVD, including modified WMH burden, CMBs burden, BG-EPVS, and CSO-EPVS.

Model 1：unadjusted; Model 2：adjusted for age and sex; Model 3：adjusted for age, sex, SBP, DBP, HR, current smoker, current drinker, hypertension, diabetes, TIA or stroke, CAD, antihypoglycemic treatment, β-blocker treatment, NIHSS score, mRS score, and qualifying events.

**Supplementary Table 6. Reclassification and discrimination statistics for HRV**

| **Variables** | **Net Reclassifcation Index(NRI)** | | **Integrated Discrimination Improvement(IDI)** | |
| --- | --- | --- | --- | --- |
|  | Estimate(95%CI),% | P value | Estimate(95%CI) | P value |
| **Presence of CSVD (Wardlaw’s scale 1-4)** | | | | |
| Basic model | Ref. |  | Ref. |  |
| Basic model+SDNN | 5.01(-2.76, 12.79) | 0.21 | 0.08(-0.02,0.18) | 0.10 |
| Basic model+RMSSD | -1.6(-9.25, 5.99) | 0.68 | 0.006(-0.02,0.03) | 0.63 |
| Basic model+SDNN+RMSSD | 5.78 (-1.99, 13.55) | 0.15 | 0.08 (-0.01,0.18) | 0.10 |
| **Presence of CSVD (Rothwell’s scale 1-6)** | | | | |
| Basic model | Ref. |  | Ref. |  |
| Basic model+SDNN | 6.21 (-2.2, 14.63) | 0.14 | 0.16 (0.01,0.30) | 0.04 |
| Basic model+RMSSD | 6.89 (-1.38, 15.16) | 0.11 | 0.18 (0.01,0.35) | 0.04 |
| Basic model+SDNN+RMSSD | 5.48 (-2.89, 13.85) | 0.20 | 0.24 (0.05,0.44) | 0.01 |
| **Moderate-to-severe CSVD (Wardlaw’s scale 2-4)** | | | | |
| Basic model | Ref. |  | Ref. |  |
| Basic model+SDNN | 3.64(-4.91, 12.19) | 0.41 | 0.01(-0.03,0.05) | 0.63 |
| Basic model+RMSSD | 3.14(-5.27, 11.56) | 0.47 | 0.08(-0.03, 0.19) | 0.15 |
| Basic model+SDNN+RMSSD | 5.78(-2.70, 14.27) | 0.19 | 0.13(-0.01, 0.27) | 0.07 |
| **Moderate-to-severe CSVD (Rothwell’s scale 2-6)** | | | | |
| Basic model | Ref. |  | Ref. |  |
| Basic model+SDNN | 7.48(-2.29, 17.26) | 0.13 | 0.02(-0.06, 0.10) | 0.61 |
| Basic model+RMSSD | 3.88(-5.74, 13.49) | 0.44 | 0.04(-0.06, 0.13) | 0.45 |
| Basic model+SDNN+RMSSD | 10.12(4.00, 19.84) | 0.043 | 0.04 (-0.06,0.14) | 0.44 |

RMSSD, the square root of the mean of the sum of the squares of differences between adjacent N-N intervals; CSVD, cerebral small vessel disease; SBP, systolic blood pressure; DBP, diastolic blood pressure; HR, heart rate; TIA, transient ischaemic attack; CAD, coronary artery disease; NIHSS, National Institutes of Health Stroke Scale score; mRS, modified Rankin scale.

RMSSD, the square root of the mean of the sum of the squares of differences between adjacent N-N intervals; SDNN, the standard deviation of all N-N intervals; CSVD, cerebral small vessel disease.

**Supplementary Table 7. Characteristics of the GWAS used in this study.**

| Phenotype | Consortium or study | Sample size | Ancestry | Genotype data | PMID |
| --- | --- | --- | --- | --- | --- |
| Exposure |  |  |  |  |  |
| RMSSD | A total of 23 cohorts | 46,952 | European | GWAS array and metabochip array | 28613276 |
| SDNN | A total of 27 cohorts | 51,975 | European | GWAS array and metabochip array | 28613276 |
| Outcomes |  |  |  |  |  |
| WMH volume | UK Biobank + CHARGE + a other WMH study | 42,310 | European | GWAS array and metabochip array | 32358547 |
| FA | UK Biobank | 17,663 | European | GWAS array and metabochip array | 32358547 |
| MD | UK Biobank | 17,467 | European | GWAS array and metabochip array | 32358547 |
| CMBs | CHARGE + UK Biobank + other cohorts | 25,862 | European | GWAS array and metabochip array | 32913026 |
| Lacunar stroke | UK DNA Lacunar Stroke studies 1 and 2 + ISGC | 254,959 | European | GWAS array and metabochip array | 33773637 |

RMSSD, the square root of the mean of the sum of the squares of differences between adjacent N-N intervals; SDNN, the standard deviation of all N-N intervals; WMH, white matter volume; FA, fractional anisotropy; MD, mean diffusivity; CMBs, cerebral microbleeds;

GWAS, genome-wide association studies; CHARGE, Cohorts for Heart and Aging Research in Genomic Epidemiology; ISGC, International Stroke Genetics Consortium.

**Supplementary Table 8. Characteristics of selected SNPs of heart rate variability**

| **Phenotype** | **SNP** | **EA** | **NEA** | **Beta** | **SE** | **EAF** | **P value** |
| --- | --- | --- | --- | --- | --- | --- | --- |
| RMSSD | rs12974991 | A | G | -0.116 | 0.008 | 0.078 | 4.57E-46 |
| RMSSD | rs10842383 | C | T | -0.065 | 0.006 | 0.862 | 2.45E-29 |
| RMSSD | rs236349 | G | A | -0.035 | 0.004 | 0.655 | 9.10E-17 |
| RMSSD | rs7980799 | A | C | -0.039 | 0.004 | 0.39 | 3.19E-20 |
| RMSSD | rs180238 | C | T | -0.034 | 0.004 | 0.333 | 7.99E-16 |
| RMSSD | rs2052015 | T | C | -0.036 | 0.006 | 0.165 | 3.56E-10 |
| RMSSD | rs36423 | T | G | -0.04 | 0.006 | 0.127 | 5.36E-11 |
| RMSSD | rs1812835 | A | C | -0.025 | 0.004 | 0.418 | 5.18E-10 |
| RMSSD | rs6123471 | T | C | -0.024 | 0.004 | 0.534 | 1.3E-08 |
| SDNN | rs12980262 | A | G | -0.06 | 0.006 | 0.076 | 2.30E-23 |
| SDNN | rs10842383 | C | T | -0.049 | 0.004 | 0.863 | 9.33E-31 |
| SDNN | rs236349 | G | A | -0.033 | 0.003 | 0.651 | 3.70E-25 |
| SDNN | rs1384598 | T | A | -0.023 | 0.003 | 0.432 | 7.37E-13 |
| SDNN | rs4262 | C | T | -0.028 | 0.003 | 0.39 | 4.26E-17 |
| SDNN | rs4899412 | T | C | -0.026 | 0.004 | 0.253 | 3.13E-13 |
| SDNN | rs2529471 | C | A | -0.021 | 0.003 | 0.429 | 1.88E-12 |
| SDNN | rs36423 | T | G | -0.033 | 0.005 | 0.129 | 6.25E-13 |
| SDNN | rs2680344 | A | G | -0.024 | 0.004 | 0.777 | 4.88E-11 |

RMSSD, the square root of the mean of the sum of the squares of differences between adjacent N-N intervals; SDNN, the standard deviation of all N-N intervals; SNPs: single nucleotide polymorphisms; EA, effect allele; NEA, non-effect allele; EAF, effect allele frequency.

**Supplementary Table 9. Mendelian randomization associations of genetic determinants of HRV parameters with the risk of CSVD**

| **Variables** | **RMSSD** | | **SDNN** | |
| --- | --- | --- | --- | --- |
|  | **β(95% CI)** | **P value** | **β(95% CI)** | **P value** |
| **WMH volume** |  |  |  |  |
| IVW | 0.88(0.72,1.08) | 0.24 | 0.91(0.70,1.19) | 0.16 |
| MR Egger | 0.74(0.48,1.15) | 0.23 | 0.60(0.25,1.43) | 0.24 |
| Weighted median | 0.85(0.67,1.07) | 0.16 | 1.00(0.71,1.40) | 0.23 |
| Simple mode | 0.78(0.55,1.11) | 0.21 | 1.09(0.63,1.88) | 0.21 |
| Weight mode | 0.81(0.62,1.05) | 0.15 | 1.06(0.62,1.78) | 0.15 |
| **FA** |  |  |  |  |
| IVW | 1.58(0.74-3.39) | 0.24 | 1.76(0.41-7.47) | 0.45 |
| MR Egger | 4.33(0.87-21.66) | 0.12 | 13.19(0.11-1631.18) | 0.35 |
| Weighted median | 1.17(0.41-3.31) | 0.77 | 0.83(0.18-3.94) | 0.82 |
| Simple mode | 1.10(0.18-6.76) | 0.92 | 0.54(0.05-5.57) | 0.63 |
| Weight mode | 0.94(0.23-3.85) | 0.94 | 0.64(0.06-7.07) | 0.73 |
| **MD** |  |  |  |  |
| IVW | 0.69(0.31-1.49) | 0.34 | 0.51(0.15-1.69) | 0.27 |
| MR Egger | 0.48(0.09-2.47) | 0.41 | 0.19(0.00-9.38) | 0.45 |
| Weighted median | 0.53(0.19-1.48) | 0.22 | 0.37(0.08-1.66) | 0.20 |
| Simple mode | 0.56(0.11-2.73) | 0.50 | 0.49(0.05-4.76) | 0.56 |
| Weight mode | 0.50(0.17-1.51) | 0.26 | 0.31(0.04-2.59) | 0.33 |
| **Lacunar stroke** |  |  |  |  |
| IVW | 0.92(0.63,1.34) | 0.66 | 0.98(0.57,1.68) | 0.94 |
| MR Egger | 1.31(0.58,2.96) | 0.54 | 1.13(0.20,6.19) | 0.90 |
| Weighted median | 1.03(0.62,1.69) | 0.91 | 1.03(0.51,2.11) | 0.93 |
| Simple mode | 1.23(0.60,2.56) | 0.59 | 1.26(0.43,3.67) | 0.69 |
| Weight mode | 1.15(0.64,2.07) | 0.65 | 1.19(0.44,3.18) | 0.74 |
| **CMBs** |  |  |  |  |
| IVW | 0.97(0.55-1.71) | 0.91 | 0.97(0.41-2.30) | 0.95 |
| MR Egger | 1.13(0.33-3.88) | 0.85 | 1.44(0.08-25.12) | 0.82 |
| Weighted median | 1.03(0.50-2.14) | 0.94 | 1.04(0.38-2.89) | 0.94 |
| Simple mode | 0.88(0.33-2.34) | 0.80 | 1.19(0.32-4.49) | 0.80 |
| Weight mode | 1.00(0.42-2.38) | 1.00 | 1.08(0.26-4.50) | 0.92 |
| **Strict lobar CMBs** |  |  |  |  |
| IVW | 1.21(0.62,2.36) | 0.58 | 1.03(0.40,2.70) | 0.94 |
| MR Egger | 2.03(0.46,9.06) | 0.38 | 6.34(0.29,139.69) | 0.29 |
| Weighted median | 1.38(0.56,3.39) | 0.48 | 1.32(0.39,4.55) | 0.66 |
| Simple mode | 1.32(0.36,4.77) | 0.68 | 1.44(0.23,9.21) | 0.71 |
| Weight mode | 1.45(0.51,4.13) | 0.51 | 1.80(0.32,10.11) | 0.53 |
| **Non-strict lobar CMBs** |  |  |  |  |
| IVW | 0.63(0.20,1.98) | 0.43 | 1.16(0.28,4.79) | 0.84 |
| MR Egger | 0.39(0.02,6.89) | 0.54 | 0.10(0.00,12.04) | 0.39 |
| Weighted median | 0.55(0.13,2.22) | 0.40 | 1.36(0.22,8.39) | 0.74 |
| Simple mode | 1.79(0.14,22.58) | 0.66 | 1.69(0.08,33.97) | 0.74 |
| Weight mode | 0.42(0.06,3.19) | 0.43 | 1.69(0.10,29.75) | 0.73 |

RMSSD, the square root of the mean of the sum of the squares of differences between adjacent N-N intervals; SDNN, the standard deviation of all N-N intervals; CSVD, cerebral small vessel disease; WMH, white matter hyperintensity; FA, fractional anisotropy; MD, mean diffusivity; CMBs, cerebral microbleeds; IVW, inverse variance weighted; 95% CI, 95% confidence intervals.

**Supplementary Figure 1. The distribution of CSVD according to the quartiles of RMSSD**

Bar plots show the distributions for (A) total burden of CSVD (Wardlaw), (B) modified total burden of CSVD (Rothwell), (C) WMH burden, (D) Presence of lacunes, (E) Presence of CMBs, (F) Brain atrophy, (G) BE-EPVS, and (H) CSO-EPVS according to the quartiles of RMSSD, respectively.

RMSSD, the square root of the mean of the sum of the squares of differences between adjacent N-N intervals; CSVD, cerebral small vessel disease; WMH, white matter hyperintensity; CMBs, cerebral microbleeds; EPVS, enlarged perivascular spaces; BG-EPVS, basal ganglia EPVS; CSO-EPVS, centrum semiovale EPVS; GCA, global brain atrophy.


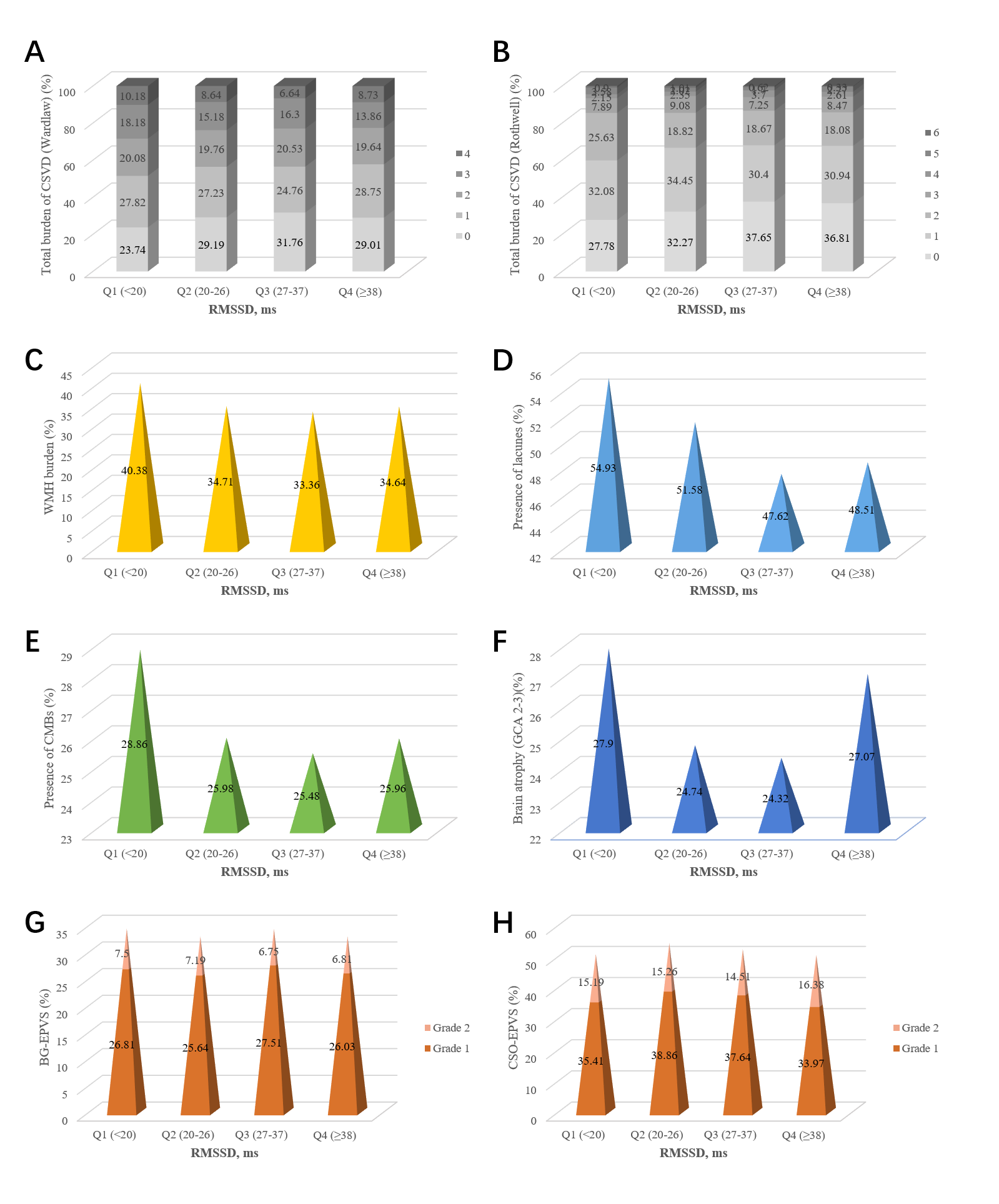


**Supplementary Figure 2. The distribution of CSVD according to the quartiles of SDNN**

Bar plots show the distributions for (A) total burden of CSVD (Wardlaw), (B) modified total burden of CSVD (Rothwell), (C) WMH burden, (D) Presence of lacunes, (E) Presence of CMBs, (F) Brain atrophy, (G) BE-EPVS, and (H) CSO-EPVS according to the quartiles of RMSSD, respectively.

SDNN, the standard deviation of all N-N intervals; CSVD, cerebral small vessel disease; WMH, white matter hyperintensity; CMBs, cerebral microbleeds; EPVS, enlarged perivascular spaces; BG-EPVS, basal ganglia EPVS; CSO-EPVS, centrum semiovale EPVS; GCA, global brain atrophy.


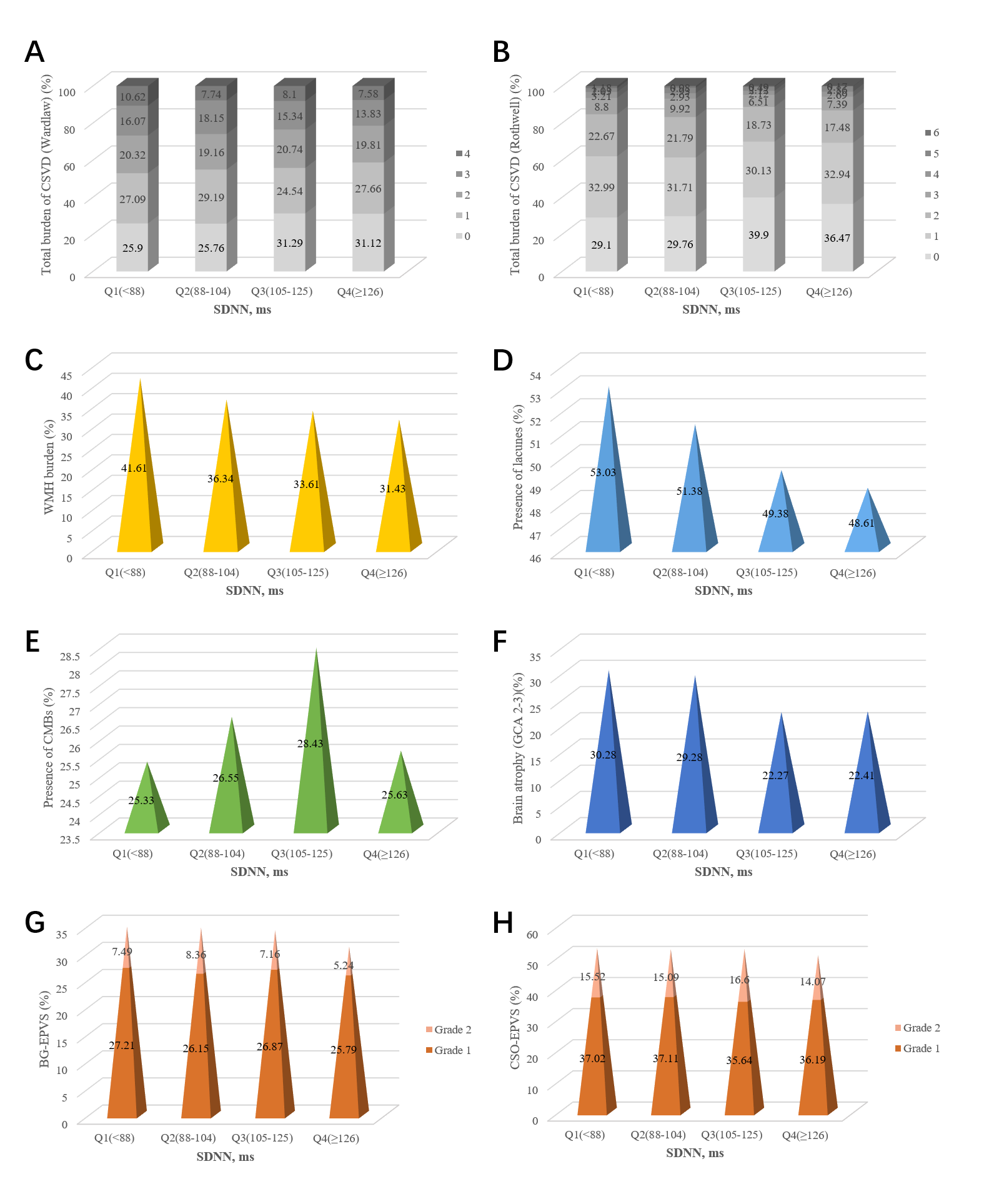

Supplement: Supplementary file 1 — Data S1: Supporting information [file CNS-29-1379-s001.docx]
